# SA-XV, a 15-amino acid fragment of host defense peptide S100A12, targets mitochondria and is protective against fungal infections

**DOI:** 10.1016/j.jbc.2025.110743

**Published:** 2025-09-18

**Authors:** Riddhi Agarwal, Karishma Biswas, Akshita Agrawal, Nisha Nandhini Shankar, Srijita Kundu, Dipanwita Roy, DeokHyun Son, Amaravadhi Harikishore, Ragothaman M. Yennamalli, DongKuk Lee, Anirban Bhunia, Sanhita Roy

**Affiliations:** 1 Prof. Brien Holden Eye Research Centre, LV Prasad Eye Institute, Hyderabad, India; 2 Dr Chigurupati Nageswara Rao Ocular Pharmacology Research Centre, LV Prasad Eye Institute, Hyderabad, India; 3 Graduate Studies, Manipal Academy of Higher Education, Manipal, India; 4 Department of Chemical Sciences, Bose Institute, Kolkata, India; 5 Department of Bioinformatics, School of Chemical and Biotechnology, SASTRA Deemed to be University, Thanjavur, India; 6 Department of Fine Chemistry, Seoul National University of Science and Technology, Seoul, South Korea; 7 SP^2^Therapeutics Inc (SP^2^TX), 105-312 Startup Center of Inst. of Molecular Biology & Genetics, Seoul National University, Seoul, South Korea; 8 School of Biological Sciences, Nanyang Technological University, Singapore, Singapore

**Keywords:** S100A12, antimicrobial peptide, *Fusarium*, mannan-binding, mitochondria, wound healing

## Abstract

Fungal infections are a huge emerging crisis, with more than 2 million people infected worldwide annually. Corneal infections caused by fungus are the major cause of vision loss and often warrant corneal transplantation. Both *Fusarium* spp. and *Candida* spp. are critical etiological agents of fungal keratitis and also common causes for invasive fungal infections with high mortality rates. In the previous work, we described growth inhibition of *Fusarium* spp. by S100A12, a host antimicrobial peptide. Here, to optimize a potential therapeutic, we have studied a 15-amino acid fragment of S100A12, SA-XV. Interestingly, SA-XV demonstrated remarkable antifungal activities, similar to the parent peptide, against both *Fusarium* spp. and *Candida* spp. SA-XV is a cell-penetrating peptide, and once internalized, it binds to fungal DNA, halts the cell cycle, and disrupts mitochondria, leading to the generation of reactive oxygen species and cell damage. The atomistic structure of the peptide determined by NMR reveals that SA-XV associates with the fungal membrane. The structural changes in SA-XV from α-helical to random coil conformation were observed in all-atom simulations. In addition, SA-XV aids in wound healing of corneal epithelial cells and attenuates the fungal burden in a murine model of fungal keratitis. Our results clearly demonstrate SA-XV as a promising antifungal candidate that targets both filamentous and nonfilamentous fungi for alternative therapeutic interventions.

Corneal infection is a leading cause of blindness worldwide and a huge socioeconomic burden (1). In India, fungal keratitis is of major occurrence, and *Fusarium* spp. and *Candida* spp. are the major etiological agents and are also included in the fungal priority pathogen list recently released by the World Health Organization (2). Both the pathogens are known to cause other forms of infections as well, and almost 2.5 million deaths occur globally every year because of fungal infections, comparable to the number of deaths from malaria or tuberculosis (3). The mounting calamity of resistance toward existing antifungals in recent years repeatedly makes the situation even worse (4). Currently available antifungal drugs have major limitations, including poor efficacy, high toxicity, and low bioavailability (5). The antifungal drug resistance is more prevalent among immunocompromised populations, often making the situation life threatening (6). The development of novel antifungal drugs that are not toxic to mammalian cells is often more challenging, as many fungal targets are closely related to the corresponding human proteins or cell structure (7). Therefore, there is a high requirement for the development of new antifungals that possess different modes of action with low toxicity and high efficacy. Antimicrobial peptides (AMPs) have been considered as the most potent alternative contenders in this context over the years.

AMPs play an important role in innate immunity present in all life domains and function as a first line of defense against various pathogen attacks (8). AMPs are cationic in nature with a net charge of +2 to +9 and contain 10 to 100 amino acids. The AMPs have an advantage over conventional antimicrobials by acting at multiple sites on the plasma membrane and intracellular targets, making it difficult for the pathogen to cope. Often, they can perform their functions in several possible modes, like membrane disruption, apoptosis induction, or internal target inhibition (9). While the primary target for most AMPs is commonly the outer membrane, many AMPs can cross the membranes and interact with cytosolic targets like nucleic acid or proteins (10). Several natural or synthetic peptides have been shown to be effective against *Candida* spp. or *Aspergillus* spp. either by disrupting the membrane or by inhibiting the cell cycle (11, 12).

Host defense peptides (HDPs) can play an important role in this aspect, as they already exist as an essential component of the host's innate immune system and often possess an immunomodulatory role in addition to their antimicrobial properties (10). Our previous study had shown that human S100A12, an HDP with two EF hand domains, binds directly to the membrane of *Fusarium* spp. causing membrane damage, and inhibits fungal growth and biofilm formation (13). Significantly increased expression of S100A12 was determined in corneal scrapings of patients with *Fusarium* keratitis (13). S100A12 also exhibited antibacterial (14, 15) and wound-healing properties both *in vitro* and *in vivo* (16). S100A12 is largely synthesized by human neutrophils (17), corneal epithelial cells (14) and corneal keratocytes (18). However, there are several obstacles for further development of S100A12 as an antifungal drug. S100A12 is a 92-amino acid protein, and often chemical synthesis of large proteins is not cost effective or nontrivial. The permeability of the large molecule across layers of cornea would also not be without challenges. Therefore, to overcome these limitations, we sought to further identify shorter fragments of the peptide that retain the antifungal functions of the parent peptide. In this study, we have identified by functional analysis a 15mer fragment (described here as SA-XV) that retained the antifungal activities of the parent peptide S100A12 and deciphered its mode of action against both filamentous *Fusarium* spp. and nonfilamentous *Candida* spp. Confocal microscopy and scanning electron microscopy revealed peptide localization in fungal cells and the membrane rupture, respectively. In addition, its efficacy was determined by topical application in an *in vivo* model of keratitis. Thus, SA-XV shows promise for development as a potent antifungal drug for combating fungal infections.

## Results

### 15mer C-terminal end of S100A12 is sufficient for antifungal activity

**Figure 1:**
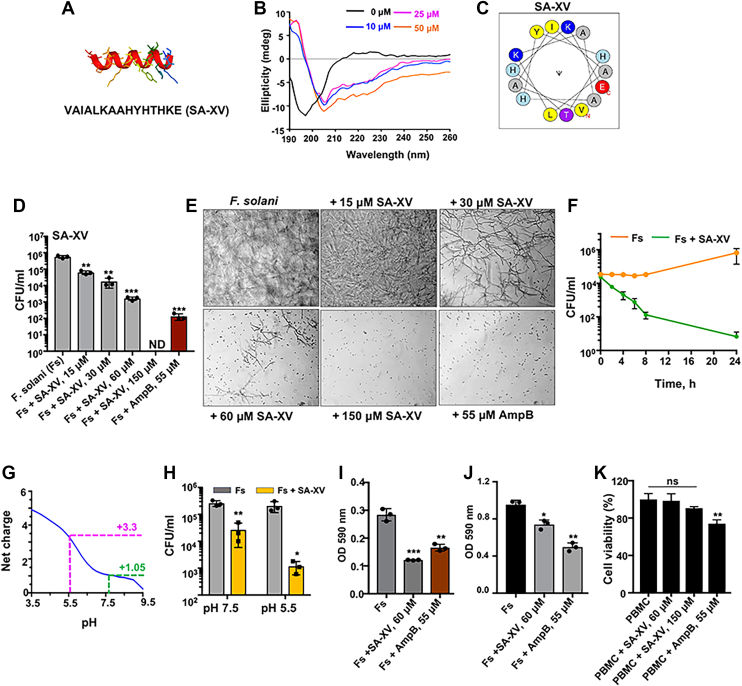
**Structure of SA-XV and its antifungal activities.** Predicted secondary structure of SA-XV (*red*) and S100A12 (*blue*) by Pep-FOLD4 and AlphaFold2, respectively (*A*), and the secondary structure of SA-XV (60 μM) in the presence of increasing concentration of SDS solution determined by CD (*B*). Helical wheel diagram of SA-XV, positively charged residues are defined by *blue color*, whereas the hydrophobic residues are *yellow* or *gray* (*C*). The growth inhibitory effect of SA-XV on *Fusarium solani* as determined by colony-forming units (CFUs) (*D*) and representative microscopic images (*E*). The kinetics of fungal growth inhibition by SA-XV was determined by CFU (*F*). Net charge of SA-XV across pH 5 to 8 shows an increase in positive charge at pH lower than 6. The theoretical net charge of the peptide was calculated using a simplified method based on the p*K*a of the free amino acids (*G*). The growth inhibition of *F. solani* by SA-XV in sodium phosphate buffer of different pH is determined by CFU (*H*). SA-XV significantly prevents biofilm formation (*I*) and acts against preformed biofilms (*J*). No cytotoxicity of SA-XV was observed against PBMC (*K*). All the experiments (*D*–*K*) were repeated at least thrice in triplicate, data are representation from a single experiment, and each point represents technical replicates. (∗ denotes *p* < 0.05, ∗∗ denotes *p* < 0.005, ∗∗∗ denotes *p* < 0.0005, and ns denotes not significant). PBMC, peripheral blood mononuclear cell.

We have earlier reported the antifungal property of full-length S100A12, an HDP, against *Fusarium* spp. and that S100A12 directly binds to the fungal membrane (13). The first reported crystal structure of S100A12 revealed a homodimer with four Ca^2+^ bound at the EF hand domains (19, 20). This study showed that His85 and His89 of one subunit form a His3Asp motif with His15 and Asp25 of the other subunit. When we sought to identify a minimal length fragment that could still retain the antifungal properties, we selected fragments consisting of the metal binding loop and/or each set of these residues that form a homodimer. One of the fragments was a 19mer (SA-XIX, sequence—VNIFHQYSVRKGHFDTLSK) involving His15 and Asp25 and the metal binding loop, and the other was a 15mer consisting of the last 15 amino acids from the C-terminal region (SA-XV, sequence—VAIALKAAHYHTHKE), which has His85 and His89 of the parent peptide, and tested their antifungal activities. The sequence of SA-XV matched that of calcitermin, which had been earlier purified from human nasal fluid as a putative C-terminal cleavage fragment of S100A12 (21). The 19mer fragment did not exhibit any antifungal activity, even at much higher concentrations (data not shown), and was not considered further. The secondary structure of the 15mer fragment, SA-XV, was predicted based on its amino acid sequences using the online Pep-Fold 4 tool (22) showing that predominantly it adopts an α-helical conformation (Fig. 1*A*). The structure was also subsequently determined by CD spectroscopy in the presence of SDS micelles, a microbial membrane mimic. We found that upon interaction with SDS, there is a deviation in the spectra from the random coil–like signature to an alpha-helical–like conformation (Fig. 1*B*). The helical wheel presentation of SA-XV indicates that residues are distributed across the helical wheel (Fig. 1*C*), the hydrophobicity of SA-XV was determined as 0.33, and the mean of the hydrophobic helical moment was 0.104. When tested for the antifungal activity, SA-XV retained the antifungal property against *Fusarium* spp. similar to the parent peptide S100A12 and significantly inhibited more than 99% of fungal growth at 60 μM (Fig. 1*D*). The microscopic images also distinctly show the reduction of fungal growth comparable to amphotericin B (AmpB) (Fig. 1*E*). In an attempt to further reduce the length, we removed the last three amino acids from this 15mer fragment and tested a resulting 12mer variant (SA-XII, sequence—VAIALKAAHYHT). However, this truncation significantly attenuated its activity, and SA-XII did not exhibit any notable antifungal property (Fig. S1*A*) at the same concentration or three times higher concentrations. Therefore, it indicates that the terminal three residues (H, K, and E) might play an essential role in the antifungal activity of the SA-XV peptide. These three amino acids of S100A12 are also conserved across several species (21).

Next, we investigated if SA-XV is also effective against nonfilamentous *Candida albicans*, another leading etiological agent for causing corneal infections worldwide (23). We found that SA-XV significantly reduced the growth of *C. albicans* by more than 95% at a concentration of 30 μM (Fig. S1*B*) and more than 99% at a concentration of 60 μM. SA-XV (60 μM) successfully reduced more than 99% of fungal growth in several ocular clinical isolates of both *Fusarium* spp. and *Candida* spp. by 24 h (Table S1) as determined by colony-forming unit (CFU). The peptide was further tested against two AmpB-resistant strains and was found to be more effective than AmpB (Fig. S1*C*).

Taken together, these results indicate that SA-XV, a 15-amino acid fragment of S100A12, exhibits significant antifungal activity against both filamentous and nonfilamentous fungi that are responsible for causing corneal infections.

### Selective activity of the peptide SA-XV toward fungal pathogens

As more than 99% reduction was observed in fungal growth by SA-XV at 60 μM, further experiments were carried out with SA-XV at this concentration. We next checked the kinetics for inhibition of *Fusarium solani* by SA-XV and found that reduction in CFU started within 2 h of peptide incubation, which decreased significantly (93.63%) by 4 h and reduced further by 99% at 24 h (Fig. 1*F*). With three histidine residues present in its sequence (almost 20% of the whole sequence), the net charge of SA-XV is significantly altered by the pH of its surrounding environment and changes from +1 (at pH 7.5) to +3.3 (at pH 5.5) (Fig. 1*G*). We summarized that this change in net charge of SA-XV may impact its antifungal efficiency. We therefore checked its antifungal effect at both pH 7.5 and 5.5 and saw increased reduction of fungal growth (from one log fold to more than three log folds) at lower pH (Fig. 1*H*); however, all the further experiments were carried out at pH 7.5, optimum for the ocular surface.

*Fusarium* spp. has the ability to form highly structured biofilms that are extremely resistant to antifungal agents compared with its planktonic form (24). We found that SA-XV (60 μM) could significantly inhibit almost 60% biofilm formation by *F. solani* comparable to that of AmpB (Fig. 1*I*). The reduction of biofilm by SA-XV was also observed by confocal microscopy (Fig. S1*D*). In addition, we checked if SA-XV could abolish preformed biofilms and found a significant reduction (*p* = 0.0056) of preformed biofilms made by *F. solani* on treatment with SA-XV (60 μM) (Fig. 1*J*). SA-XV also significantly inhibited more than 70% biofilm formation by *C. albicans* (Fig. S1*E*).

In contrast to its action against fungi, SA-XV did not show any substantial cytotoxicity toward peripheral blood mononuclear cells (Fig. 1*K*), human corneal epithelial cells (HCECs) (Fig. S2*A*), or sinonasal epithelial cells (Fig. S2*b*), even at higher concentrations, as determined from a metabolic 3-[4,5-dimethylthiazol-2-yl]-2,5 diphenyl tetrazolium bromide assay. The hemolytic activity toward human erythrocytes was also checked, and no significant hemolysis of red blood cells (Fig. S2*C*) was observed compared with the untreated control. The selectivity of the peptide was further checked using HCEC as a model that mimics corneal infection *in vitro.* HCECs were infected with *F. solani* in the presence or absence of SA-XV, and cell cytotoxicity was checked by propidium iodide (PI) staining and lactate dehydrogenase assay. Increased cell death was observed on *F. solani* infection (Fig. S2*D*ii), which was reduced significantly in the presence of SA-XV (Fig. S2*D*iii). SA-XV alone (Fig. S2*D*iv) had comparable effect as control cells (Fig. S2*D*i). A similar result was observed by the lactate dehydrogenase assay from the supernatant collected from these cells (Fig. S2*E*). We next checked the selectivity index (CC_50_/MIC_90_) of SA-XV, often used to denote the cell selectivity of antimicrobial therapeutic agents (25). The MIC_90_ of SA-XV for *Fusarium* and *Candida* is 15 μM, and IC_50_ value for cell cytotoxicity was found to be 302.1 μM by 3-[4,5-dimethylthiazol-2-yl]-2,5 diphenyl tetrazolium bromide assay against HCEC; therefore, the calculated selectivity index of SA-XV was 20.17, indicating the peptide to be 20 times more selective toward fungus than HCECs. We further checked if the peptide has any irritation effect using the hen's egg chorioallantoic membrane assay (26) and found the peptide to be nonirritant (Fig. S2*F*) compared with 0.1% NaOH used as a positive control. The stability of the peptide was also checked after subjecting it to different conditions as mentioned (Fig. S2*G*); similar retention times were obtained for all the conditions along with similar elution profiles (not shown). The stability of the peptide was further checked in the presence of fetal bovine serum (27) at 37 °C and was found to be stable with a half-life of over 180 min (Fig. S2*H*). These data clearly indicate that the peptide is selective against fungus compared with mammalian cells and stable under various mentioned conditions.

### SA-XV binds to mannan in the cell wall and to phospholipids of *Fusarium* spp. and *Candida* spp

**Figure 2:**
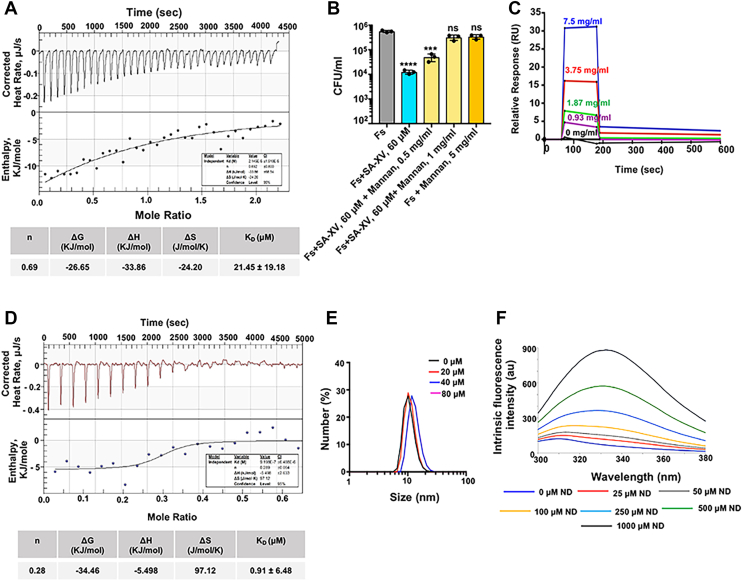
**SA-XV binds to the fungal cell wall.** The binding of SA-XV to live *Fusarium solani* was determined by ITC and appears to be exothermic in nature. The table beneath the figure lists thermodynamic parameters at 310 K, n denotes binding stoichiometry (*A*). The binding of SA-XV to mannan present on the fungal cell wall was determined by competitive binding assay by quantifying CFU (*B*) and surface plasmon resonance (*C*). The interaction of SA-XV with nanodiscs (NDs) representing the fungal membrane was determined by ITC. The thermodynamic parameters are listed in tabular form below (*D*). Dynamic light scattering (DLS) of NDs titrated with increasing concentrations of SA-XV indicated significant changes in size of the NDs (*E*). Fluorescence emission shifts of tyrosine (λ_max_) in SA-XV in the presence of NDs. The observed shift in spectra indicates a direct interaction between the peptide NDs (*F*). (∗ denotes *p* < 0.05, ∗∗ denotes *p* < 0.005, ∗∗∗ denotes *p* < 0.0005, and ns denotes not significant). CFU, colony-forming unit; ITC, isothermal titration calorimetry.

Next, we wanted to explore the mode of action of SA-XV against fungus and if the peptide binds to the fungal cell wall. HDPs often first interact with the fungal cell wall to exert their antifungal actions. For that, we first performed an isothermal titration calorimetry assay to determine the thermodynamic parameters guiding the binding interactions of SA-XV with live *Fusarium* spp. The interaction was observed to be exothermic in nature (Fig. 2*A*). A negative value for the Gibb’s free energy, ΔG (−26.65 kJ/mol), clearly indicated spontaneous and thermodynamically favorable interaction. Other binding parameters like the change in enthalpy (ΔH) as well as the change in entropy (ΔS) were also calculated, and the values were −33.86 kJ/mol and 24.20 J/mol/K, respectively. The dissociation constant (*K*_*D*_) for the peptide with *Fusarium* was 21.45 ± 19.18 μM, which indicated moderate binding affinity of the peptide to the fungus. The cell wall of both *Fusarium* and *Candida* is made up mostly of mannan and chitin along with β-(1,3) glucan (28, 29). We sought to determine if SA-XV binds to mannan and that the binding has a correlation with antifungal activity; a competition assay was performed. SA-XV was first preincubated with different concentrations of mannan at 4 °C for 2 h and then tested for its efficiency to inhibit fungal growth. We found significant reduced inhibition of growth for both *Fusarium* (Fig. 2*B*) and *Candida* (Fig. S3*A*) by SA-XV (60 μM) when bound to an increasing amount of mannan. This indirectly shows that SA-XV binds to mannan, and the mannan-bound peptide was therefore unable to inhibit fungal growth. This was further confirmed by the surface plasmon resonance (SPR) experiment. We observed 1:1 binding between mannan and SA-XV with a *K*_*D*_ value of 1.78 μM (Fig. 2*C*). The interaction of mannan with SA-XV was also put through a blind docking experiment using the DockThorv2 program and validated by PyMOL (Schrödinger, LLC). The best model showed a negative value of affinity energy (−7.45 kcal/mol), indicating the binding to be spontaneous (Fig. S3*B*). Polar bonds were detected between mannan and residues Lys6 and His11 of SA-XV. However, no substantial binding was observed by SPR between SA-XV and galactomannan (data not shown), which is the main component present in the cell wall *of Aspergillus* spp. (30)*,* another common etiological agent causing keratitis. We also checked the effect of the peptide on *Aspergillus flavus* and found SA-XV failed to inhibit the growth of *A. flavus* (Fig. S3*C*). This can be possibly because of the inability of the peptide to bind to galactomannan, indicating the selectivity of SA-XV and that binding of the peptide to the fungal cell wall might be the first crucial step for growth inhibition.

Several AMPs are known to bind to the phospholipids present on the fungal membrane along with S100A12 (13, 31), which lies beneath the cell wall. We next determined the membrane phospholipid that SA-XV might bind to on the fungal membrane by protein–lipid overlay assay. SA-XV was found to strongly bind to phosphatidylinositol, PI3P, PI4P, PI5P, and cardiolipin (CL) and weakly to phosphatidylethanolamine (PE) and sulfatide (Fig. S3*D*) but not to cholesterol (CHL) that is found predominantly in mammalian membranes. In an attempt to further understand the interaction of the peptide with the fungal membrane, we used nanodiscs (NDs), discoidal lipid layers mimicking the native fungal membrane. The NDs were composed of dipalmitoylphosphatidylcholine, dipalmitoylphosphatidylethanolamine, and ergosterol (ERG) in a 5:4:3 ratio that mimics the fungal membrane and checked the binding affinity of the peptide with the NDs. We found that the peptide binds with the ND favorably with a *K*_*D*_ value of 0.91 ± 6.48 μM (Fig. 2*D*) in a spontaneous and entropy-driven process. The interaction of SA-XV with the NDs was further assessed by dynamic light scattering measurements, an efficient way to assess the effect of the peptide on the size distribution of the NDs. We observed an increase in hydrodynamic diameter of the NDs with increased concentration of SA-XV from 10.3 nm to 18 nm, indicating that the peptide causes deformations in the lipid environment of the NDs, which further leads to aggregation and formation of larger sized particles in the aqueous solution (Fig. 2*E*). We also checked the effect of the peptide on NDs by transmission electron microscopy and observed the loss of the distinct circular shape of the NDs and generation of larger aggregates in the peptide-treated NDs (Fig. S3*E*).

Next, we explored the intrinsic tyrosine fluorescence for understanding the interaction of SA-XV with NDs mimicking the fungal membrane. Changes in the intrinsic fluorescence usually are an indication of the changes in the microenvironment of the Tyr residue (Y10), in the presence of the lipid environment of the NDs. We observed a red shift in the emission maxima of the peptide of up to 20 nm on the addition of increasing concentrations of the NDs (Fig. 2*F*), suggesting the probable changes in the solvent exposure of the Tyr residue in the presence of the NDs. In addition to the changes in fluorescence intensity, sigmoidal dependence of the shift was evident at all the ND compositions (Fig. S3*F*). The aforementioned data clearly indicate that the terminal 15 residues at the C-terminal region of S100A12 are sufficient to interact with the fungal cell wall and phospholipid membrane of both *Fusarium* and *Candida*.

### SA-XV translocates into fungal cells in an energy-dependent process and targets the nucleus and mitochondria

**Figure 3:**
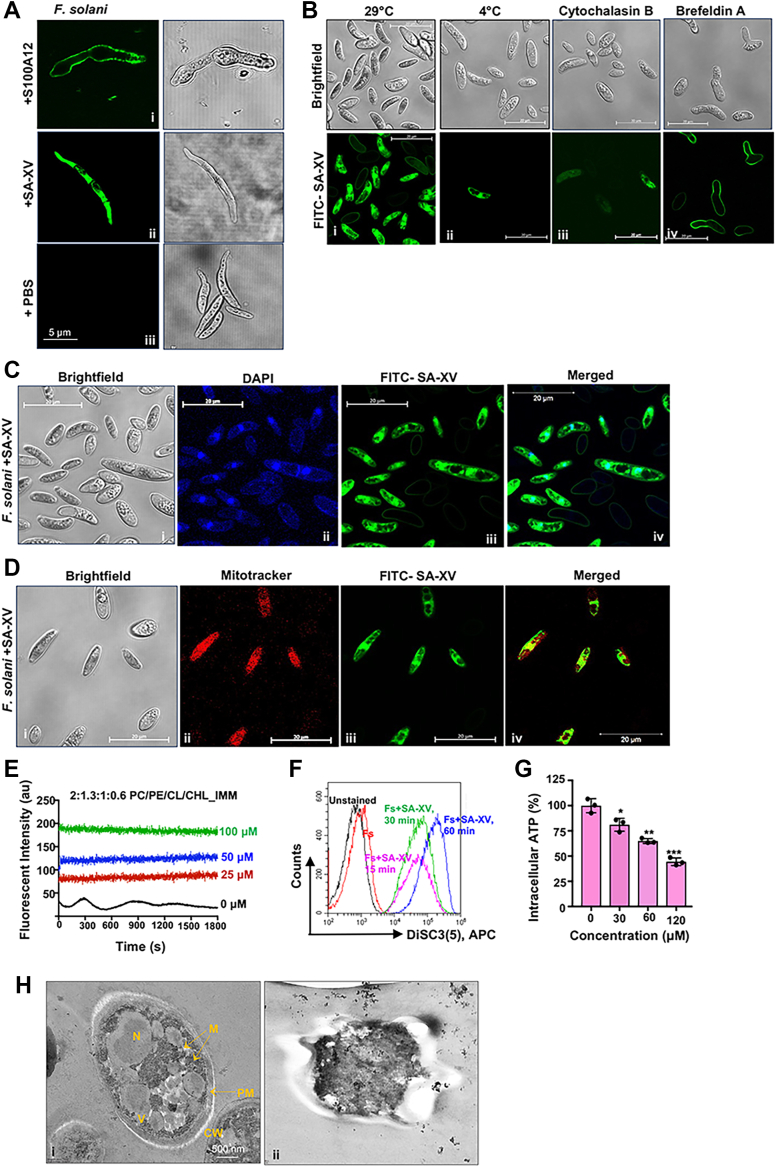
**SA-XV translocates into the fungal cytoplasm.** Confocal microscopy of *Fusarium solani* treated with SA-XV conjugated with FITC (*green*) (*A*) and uptake of FITC-SA-XV under different conditions (*B*). Confocal microscopy images of colocalization of FITC-SA-XV (*green*) and nucleus (*blue*) (*C*) and FITC-SA-XV (*green*) and mitochondria stained with MitoTracker (*red*) (*D*). Images are representative of at least three independent experiments. Calcein dye leakage assay using artificial liposome made of PC/PE/CL/CHL treated with SA-XV (60 μM) (*E*). SA-XV causes disruption of membrane potential in *F. solani*, which was detected using 3,3′-diprophylthiadicarbocyanine iodide and determined by flow cytometry (*F*). The intracellular ATP level of *F. solani* in the presence of different concentrations of SA-XV was determined by luciferase assay (*G*). The transmission electron microscopic image of *F. solani* in the presence or absence of SA-XV for 6 h (*H*). All experiments have been done at least thrice in duplicates, data are representation from a single experiment, and each point represents technical replicates. (∗ denotes *p* < 0.05, ∗∗ denotes *p* < 0.005, ∗∗∗ denotes *p* < 0.0005, and ns denotes not significant). CHL, cholesterol; CL, cardiolipin; PC, phosphatidylcholine; PE, phosphatidylethanolamine.

We had earlier reported that S100A12 directly binds to the fungal membrane (Fig. 3*A*i) (13) and causes membrane disruption. To determine the mode of action of SA-XV further, both *Fusarium* and *Candida* were incubated with SA-XV for 2 h and observed under the microscope. We found that SA-XV translocates into both *Fusarium* (Fig. 3*A*ii) and *C. albicans* (Fig. S4*A*), and the translocation happens as early as within 30 min (Fig. S4*B*). Next, to explore whether the mode of translocation involved endocytosis, uptake of the peptide into the fungal cells was studied under conditions like low temperature that inhibits all potential endocytic processes or in the presence of an inhibitor that inhibits energy-mediated processes. The translocation of SA-XV (60 μM) was reduced significantly at 4 °C compared with that at 29 °C (Fig. 3*B*i and ii), and in the presence of cytochalasin B (5 μM), an inhibitor of actin polymerization and the endocytosis process (32) (Fig. 3*B*iii). The peptide uptake was also appreciably reduced in the presence of brefeldin A (5 μM), a toxin that inhibits endocytosis and vesicle trafficking (33) (Fig. 3*B*iv). These prove unambiguously that SA-XV is cell penetrating in nature and capable of reaching intracellular compartments of fungal cells by an endocytosis-dependent mechanism. We next checked if modification of the cell wall affected the translocation of SA-XV. After removal of the glycosylated proteins present on the outer fungal layer by proteinase K (0.5 mg/ml), there was reduced uptake of SA-XV into the fungal cells (Fig. S4*C*).

Since the peptide translocates into the fungal cytoplasm, we next determined the intracellular localization of the peptide. We observed that SA-XV colocalizes with the nucleus of the fungal cells (Fig. 3*C*) as evident from the overlapping of blue and green fluorescence (Fig. 3*C*iv). We have earlier seen that SA-XV binds to CL, a lipid present predominantly in the inner mitochondrial membrane (IMM). Therefore, we sought to determine the effect of the peptide on fungal mitochondria as well. We observed that SA-XV colocalizes within the mitochondria as indicated by the overlay of fluorescence staining of the peptide (*green*) and the mitochondria (*red*), which was detected by the MitoTracker (Fig. 3*D*). We further observed calcein dye leakage from the artificial liposomes made up of CL that mimics IMM composed of phosphatidylcholine/PE/CL/CHL (2:1.3:1:0.6) by SA-XV (Fig. 3*E*) and also detected the disruption of mitochondrial membrane potential in *F. solani* within 30 min by SA-XV (Fig. 3*F*) using fluorescent voltage reporter 3,3′-dipropylthiadicarbocyanine iodide. The depolarization results in energy limitations, and therefore, intracellular ATP levels in *F. solani* were determined using a luciferase assay. We observed more than a 30% reduction in intracellular ATP content in SA-XV-treated cells at a 1× MIC, compared with untreated *Fusarium* (Fig. 3*G*), indicating that SA-XV has a definite impact on energy metabolism. We next aimed to observe the organelle morphology of *Fusarium* in the presence of SA-XV. While the control fungal cells showed intact nuclei and mitochondria (Fig. 3*H*i), the treated fungal cells were completely damaged, and the organelles could not be clearly identified (Fig. 3*H*ii). These results evidently establish that the plasma membrane, nucleus, and mitochondrial inner membrane are foremost targets of SA-XV. Interestingly, we observed SA-XV could penetrate into HCECs to some extent, but no colocalization of the peptide was noted within the mammalian nucleus or mitochondria (Fig. S4*D*) adding to the selectivity of the peptide.

### SA-XV causes cell cycle arrest at the G0/G1 phase in fungal cells, binds to DNA, and inhibits *in vitro* transcription

**Figure 4:**
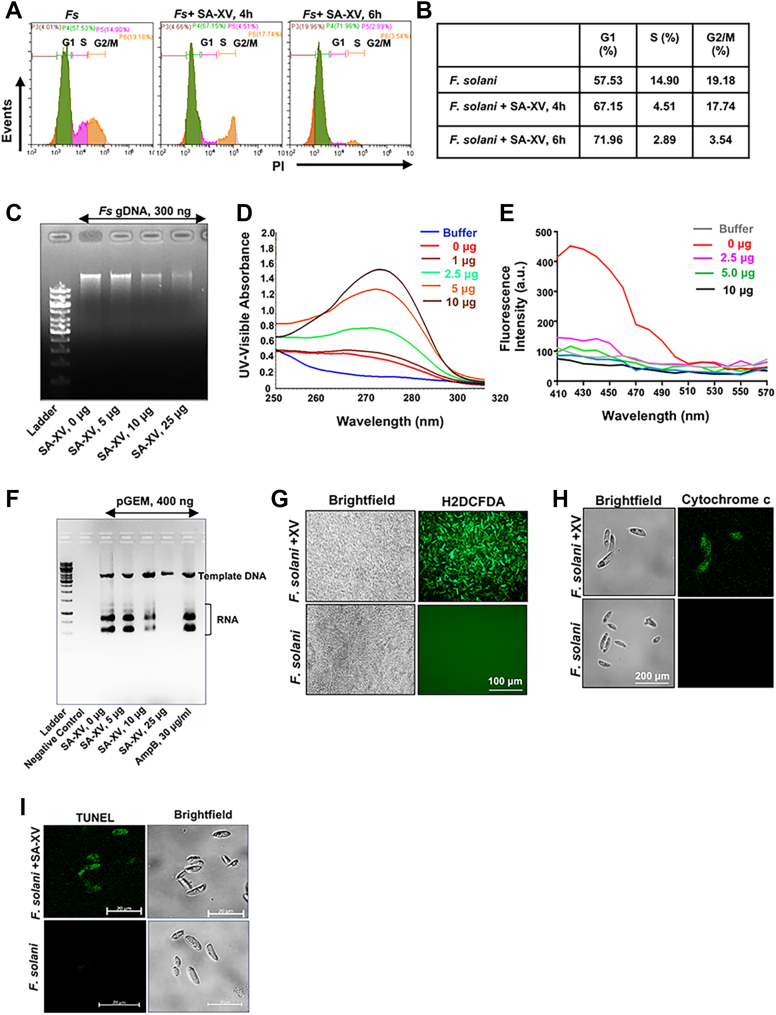
**Interaction between DNA and SA-XV and its inhibitory effects on bacterial transcription.** The effect of SA-XV (60 μM) on progression of cell cycle in *Fusarium solani* as determined by flow cytometry (*A*) and representation of percentage of cells in each phase in tabular form (*B*). The binding experiment of SA-XV (0–25 μg) with Fusarium genomic DNA (300 ng) was assessed by agarose gel retardation experiment (*C*). The binding was also determined by ultraviolet spectral assay over a wavelength range of 220 to 320 nm (*D*). DAPI competitive binding assay was performed by recording the fluorescence spectra from 400 to 600 nm (excitation = 358 nm) with sampling interval set to 10 nm (*E*). RNA synthesis in the presence of different concentrationsof SA-XV or amphotericin B was obtained by *in vitro* transcription assay and run on agarose gel. A reaction without DNA template (pGEM) was used as a negative control (*F*). SA-XV induced ROS generation in *F. solani* as determined by fluorescence microscopy (*G*). The expression of cytochrome *c* was determined in *F. solani* exposed to SA-XV by fluorescence microscopy using anti–cytochrome *c* antibody followed by Alexa Fluor 488 secondary antibody (*H*). The breakage in DNA was determined by TUNEL assay (*I*). Images are representative of at least two independent experiments done in duplicates. DAPI, 4′,6-diamidino-2-phenylindole; ROS, reactive oxygen species.

We found SA-XV to colocalize in the nucleus and predicted that the interaction might cause obstruction to vital processes like replication or transcription, leading to cell death. Therefore, we checked if SA-XV influences cell cycle progression in *Fusarium* spp. and *Candida* spp. and investigated cell cycle distribution by flow cytometry (Beckman Coulter). We found an increased proportion of cells in the G0/G1 phase, indicating that SA-XV had induced cell cycle arrest in the G0/G1 phase in both *F. solani* (Fig. 4, *A* and *B*) and *C. albicans* (Fig. S5*A*) compared with untreated fungal cells. Concurrently, the proportion of cells reduced in both the S and G2/M phases. The G1 arrest usually suggests damaged DNA of the cells and a window to allow repair before duplication of damaged DNA occurs and indicates inhibition of transcription or replication by directly binding to DNA. Therefore, we next investigated the binding of SA-XV and genomic DNA (gDNA). Fungal gDNA was incubated with an increasing amount of SA-XV (5–25 μg) for 4 h, and reduced intensity of the gDNA bands was observed in the agarose gel (Fig. 4*C*), suggesting the formation of a stable peptide–DNA complex. This observation was further confirmed by ultraviolet spectra assays and fluorescence quenching assays. There was an increase in absorbance, a hyperchromic shift, with an increasing concentration of the peptide (Fig. 4*D*), indicating the binding of the peptide to the DNA. We then performed the docking of SA-XV with gDNA of *Fusarium* (Protein Data Bank [PDB] ID: 4EFJ) and observed that the peptide binds to the major groove of the DNA (Fig. S5*B*) with a predicted binding energy of −1.78 kcal/mol, and hydrogen bonds (shown in *yellow lines*) were formed between DNA and Tyr, Lys, and His residues of the peptide. Next, we performed the competitive binding assays with both ethidium bromide (EtBr), known to intercalate with DNA or 4′,6-diamidino-2-phenylindole (DAPI), which binds to the minor groove of DNA, which causes an increase of its fluorescence intensity (34). While we observed a slight reduction in the fluorescence intensity by EtBr bound to DNA with increasing concentration of the peptide (Fig. S5*C*), a significant reduction of fluorescence intensity by DAPI bound to DNA was noted (Fig. 4*E*), indicating an interaction between the peptide and DNA, possibly because of displacement of DAPI by the peptide. These data clearly imply that SA-XV binds to DNA in a groove instead of intercalation. We further hypothesized that this binding of the peptide with DNA could subsequently inhibit the transcription process, leading to cell death. To confirm this hypothesis, we performed an *in vitro* transcription assay using T7 RNA polymerase, plasmid DNA, and increasing amounts of SA-XV. The newly synthesized RNA was run in agarose gel, and a reduced amount of newly synthesized RNA was observed with increasing SA-XV (Fig. 4*F*). AmpB, a commonly used antifungal, was used as a positive control and did not show any appreciable effect. The cell cycle arrest is also known to be associated with excessive reactive oxygen species (ROS) generation and apoptosis (35). We therefore determined if SA-XV induced ROS generation similar to the parent peptide and mediated apoptosis as a mode of fungal cell death. SA-XV induced increased ROS generation in *F. solani* as observed under a fluorescent microscope (Fig. 4*G*). We next checked the release of cytochrome *c* in *Fusarium* in response to SA-XV. Cytochrome *c* is a key regulator of apoptosis in eukaryotes and is released from mitochondria under cell death conditions like increased ROS (36). Increased cytochrome *c* staining was observed in *Fusarium* exposed to SA-XV by 2 h (Fig. 4*H*), and DNA fragmentation was identified by TUNEL assay (Fig. 4*I*), which fluorescently label strand breaks and is a direct method of DNA damage measurement (37), indicating apoptosis taking place. Taken together, these findings show that the peptide SA-XV binds to fungal DNA and inhibits transcription that leads to cell death *via* apoptosis.

### SA-XV forms pores in the fungal membrane during translocation

**Figure 5:**
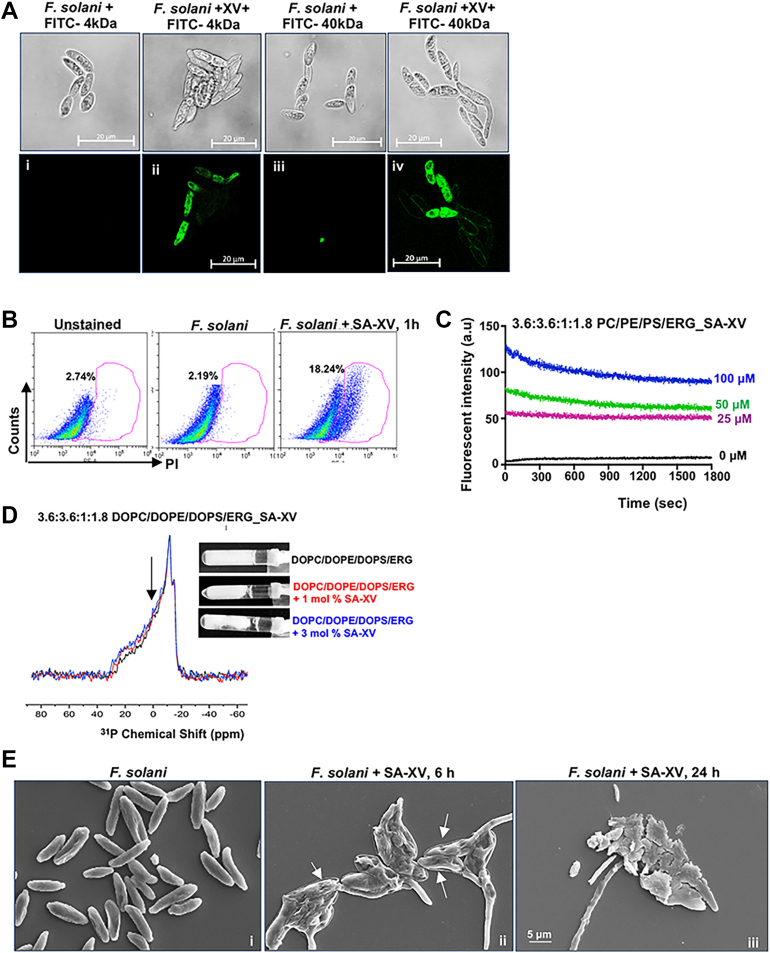
**SA-XV forms pore and causes damage to the fungal membrane.** The formation of pores in fungal membrane by SA-XV was determined using FITC-dextran of different molecular weight by confocal microscopy in *Fusarium solani* (*A*). The membrane damage was detected by determining the uptake of propidium iodide by flow cytometry (*B*), which was further checked by calcein dye leakage assay using artificial liposomes made of PC/PE/PS/ERG (*C*). 31P solid-state NMR spectra of DOPC/DOPE/DOPS LUVs incorporated ERG (*left*), samples were treated with 0 mol % (*black*), 1 mol % (*red*), and 3 mol % (*blue*) SA-XV. For the latter case, pictures (*inset*) showed the LUV samples after the addition of the peptide, and aggregation of the LUVs is clearly seen. *Arrows* indicate the changes upon addition of peptide to the DOPC/DOPE/DOPS/ERG LUV (*D*). Scanning electron microscope images of *F. solani* treated with SA-XV (60 μM) at 6 and 24 h (*E*). DOPC, dipalmitoylphosphatidylcholine; DOPE, dipalmitoylphosphatidylethanolamine; DOPS, 1,2-dioleoyl-*sn*-glycero-3-phospho-l-serine; ERG, ergosterol; LUV, large unilamellar vesicle; PC, phosphatidylcholine; PE, phosphatidylethanolamine; PS, phosphatidylserine.

Next, we wanted to explore if SA-XV forms pores of distinct size in the membrane during translocation and also causes membrane breaks. Both *Fusarium* (Fig. 5*A*) and *Candida* (Fig. S6*A*) were incubated with SA-XV for 2 h in the presence of FITC–dextran (FD) of lower (4 kDa) and higher (40 kDa) molecular weight and were observed under a confocal fluorescence microscope. The diameter of FD 4 is 2.8 nm and that of FD 40 is 9 nm, which would give an idea of the pore size if the dextran enters fungal cells. We found that the uptake of FD 4 was almost 50% to 60% (Fig. 5*A*ii) and that of FD 40 was around 30% (Fig. 5*A*iv) in both *Fusarium* and *Candida* (Fig. S6*A*). No uptake of FD of either size was observed in fungus not exposed to SA-XV (Fig. 5*A*i and iii). This clearly demonstrated that the peptide forms pores of larger than 9 nm on the fungal membrane, possibly through a toroidal pore mechanism similar to earlier reported peptides during translocation (38).

The formation of pores was further confirmed by the uptake of nucleic acid stain PI, which permeates the fungal cell membrane when compromised, as seen by flow cytometry (Fig. 5*B*) and also by fluorescence microscopy in *Fusarium* spp. (Fig. S6*B*). The uptake of PI was also seen in *Candida* (Fig. S6*C*) by fluorescence microscopy, indicating the formation of pores by SA-XV. To further confirm the membrane damage, we examined the calcein dye leakage from artificial liposomes made of phosphatidylcholinephosphatidylcholine, PE, phosphatidylserine, and ERG, which mimicked the fungal membrane, on incubation with SA-XV. Increased dye leakage was noted with increased concentration of SA-XV (Fig. 5*C*), indicating that the peptide binds to fungal lipids and causes membrane damage. In addition, ^31^P solid-state NMR spectra of multilamellar vesicles (MLVs) composed of dipalmitoylphosphatidylcholine/dipalmitoylphosphatidylethanolamine/1,2-dioleoyl-*sn*-glycero-3-phospho-l-serine/ERG with a molar ratio of 3.6:3.6:1:1.8 showed a typical powder pattern spectra around −10 ppm, and the relative intensity of the parallel edges increased with the addition of SA-XV (Fig. 5*D*). The chemical shift anisotropy spans were 41.6 ppm, 44.6 ppm, and 46.4 ppm for the peptide-free, 1 mol%, and 3 mol% peptide, respectively. The shifts around 30 ppm for the phospholipid molecules parallel to the external magnetic field are clear in the spectra. These data suggest that the spherical shape of large unilamellar vesicles (LUVs) was deformed or aggregated in the presence of SA-XV, as seen in the picture inset, and the orientation of the phospholipid headgroup changed. No significant uptake of PI was, however, observed in HCECs incubated with SA-XV (60 μM) for 6 h compared with untreated cells (Fig. S6*D*), indicating the peptide to be specific for the fungal membrane and not causing any damages to the mammalian cell membrane. The effect of SA-XV on membrane morphology of *Fusarium* was further examined by scanning electron microscopy. The disruption on different parts of the fungal membrane along with pore formation was observed (shown by an *arrow*) by 6 h (Fig. 5*E*ii) compared with untreated *Fusarium* (Fig. 5*E*i), whereas the membrane completely got dissolved/ruptured by exposure to the peptide for 24 h (Fig. 5*E*iii). SA-XV also caused complete membrane damage in *Candida* by 6 h (Fig. S6*E*ii) compared with untreated *Candida* (Fig. S6*E*i).

### Elucidation of the 3D structure of SA-XV and its interaction with the fungal membrane

**Figure 6:**
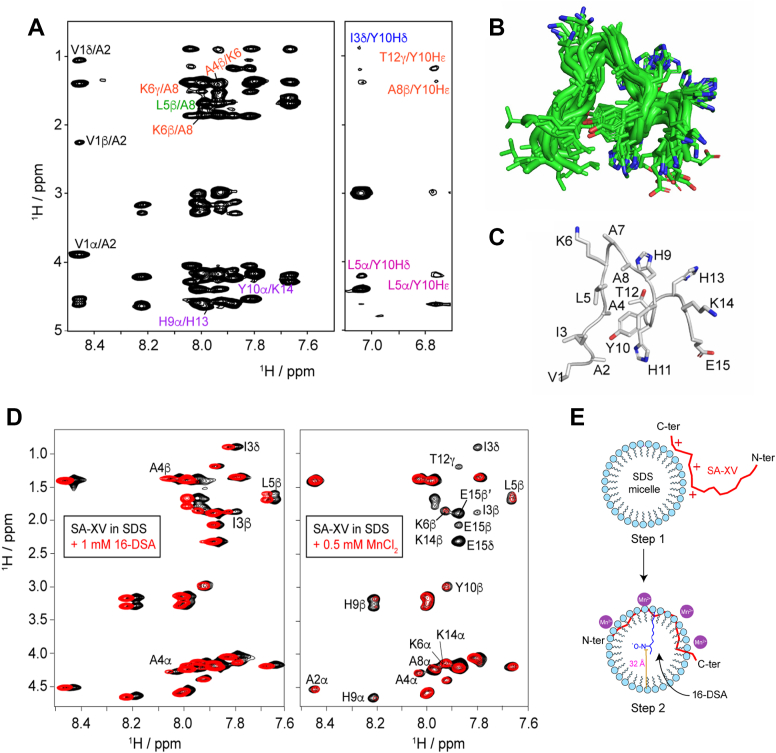
**Structural elucidation of SA-XV in the presence of SDS micelle.** NOESY spectra depicting medium- and long-range NOE connectivities for the SDS micelle–bound form of SA-XV (*A*). Twenty ensemble lowest energy structures of SDS micelle–bound SA-XV (*B*). *Cartoon representation* of a single molecule of SA-XV bound to SDS micelle, highlighting the orientation of each residue of the peptide (*C*). TOCSY spectra of SA-XV in 200 mM SDS micelles after addition of 1 mM 16-DSA (*left panel*) and 0.5 mM MnCl_2_ (*right panel*) at 310 K (*D*). Schematic representation of a single SA-XV peptide in SDS micellar environment generated in Biorender.com (*E*). 16-DSA, 16-doxyl-steric acid.

Solution-state NMR spectroscopy was used to gain an insight into the structural aspects of SA-XV in the presence of SDS micelles, a model mimicking the negatively charged microbial membrane. SDS micelles have been utilized extensively for structural elucidation of peptides using high-resolution NMR spectroscopy because of the rapid tumbling motion of the peptide–micelle association in solution (39). Therefore, to obtain the atomic resolution of the peptide's conformation, we acquired the 2D ^1^H–^1^H NOESY spectra in the presence of SDS micellar environments, assigned, and calculated the structure of SA-XV. We observed the lack of medium- to long-range NOEs of SA-XV in aqueous solution, signifying that the structure is mostly flexible and does not adopt a well-defined conformation in the aqueous solution. On the other hand, in the presence of SDS, we obtained a sufficient number of sequential and medium-range NOE crosspeaks in the NOESY spectra, indicating the change in structural conformation (Fig. 6*A*). In addition to sequential C^α^H/NH (i to i +1) NOEs, some medium-range C^α^H/NH (i to i + 2) NOEs were observed in the sequence of SA-XV between A4/K6 and K6/A8. Very few long-range NOEs, i to i + 3 (L5/A8) as well as i to i + 4 (H9/H13, Y10/K14), were also obtained (Fig. S8, *A* and *B*). Several aromatic/aliphatic NOE connectivities were observed. The tyrosine phenyl ring protons (Y10) are in close proximity to methylene and methyl protons of neighboring aliphatic residues (Fig. 6*A*). We obtained minor deviations from the chemical shifts of random coil conformation for all the residues from the secondary chemical shift plot of SA-XV, indicating the adaptation of a secondary structure (Fig. S8*C*). Lack of CαH/CαH as well as long-range NH/NH contacts negated the presence of antiparallel β-sheet conformation. Using NOE-based distance constraints, we determined 20 ensemble structures of SDS micelle–bound SA-XV with an RMSD value of 1.66 (Fig. 6*B*). Close inspection into the 3D structure of SA-XV in SDS reveals that it is flexible mostly at the C-terminal end (Fig. 6*C*). The backbone dihedral angles (Φ, Ψ) were mainly assembled in the most favorable regions and additionally allowed sections in the Ramachandran plot (Table S3). In the SDS micellar environment, SA-XV adopts a weakly bound structure. The positively charged His residues (H9, H11, and H13) mainly participate in electrostatic interaction with the negatively charged micellar head groups. The interactions between Y10 and I3, Y10 and A8, Y10 and T12, as well as Y10 and L5 facilitate cation–Π interaction as well as CH_3_–aromatic (Π) interaction among the residues.

A paramagnetic relaxation enhancement experiment was performed with two paramagnetic quenchers—16-doxyl-steric acid (16-DSA) and MnCl_2_—used to gain insights into the specific localization of SA-XV in SDS micellar systems. 16-DSA usually penetrates the micelles, positioning themselves near the alkyl chains of the lipids and induces relaxation of the residues in close proximity to it. No significant attenuation was observed even in concentrations as high as 1 mM 16-DSA (Fig. 6*D*). On the other hand, an addition of MnCl_2_ attenuates signals of the residues, which are weakly bound to the SDS micelle, and are exposed to the solvent. At a concentration of 0.5 mM MnCl_2_, significant attenuation (>50%) of C-terminal residues (H9, Y10, T12, K14, and E15) was observed. Side chains of I3, L5, and K6 were also attenuated to a great extent (Fig. 6*D*). Therefore, we conclude that SA-XV remains weakly bound on the surface of the SDS micelle, with the C terminal remaining flexible and exposed to the solvent (Fig. 6*E*). The 3D structure of SA-XV bound to SDS micelle has been deposited in the PDB. The PDB accession code of the structure is 9U7M.

**Figure 7:**
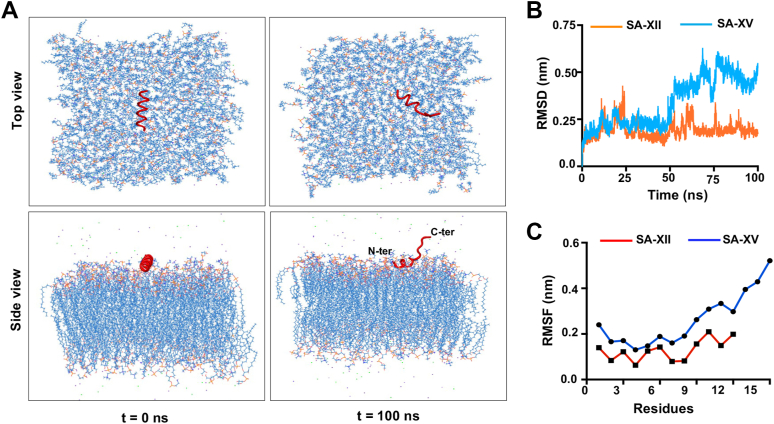
**Molecular dynamics simulation of SA-XV with fungal membrane.** Snapshots of the top and side views of SA-XV interacting with the fungal phospholipid at 0 and 100 ns (*A*). The graphical representation of comparison of RMSD values (*B*) and RMSF values (*C*) between SA-XV and SA-XII. RMSF, root mean square fluctuation.

We next carried out all-atom molecular dynamics (MD) simulations of the AlphaFold2-predicted 3D structures of SA-XII and SA-XV to investigate how they interact with the model fungal membrane; a single copy of each peptide was simulated in horizontal orientation with the plasma membrane. These setups were run for 100 ns each to monitor the approach and interaction of the peptide with the membrane. The conformational change is evident in SA-XV as it initiates to interact with the membrane, and almost half of the peptide is unfolded. The initial and final snapshots of the peptide-membrane simulations are shown (Fig. 7*A*) both from top and side views. The video of the interaction of SA-XV with the lipid bilayer is shown in Video S1, which clearly shows the unfolding of the peptide at the C-terminal region on interaction with the designed membrane. In contrast, almost no change was observed in the conformation of SA-XII on membrane interaction (Fig. S7*A*). The structural changes that the peptide underwent during simulation are evidenced by the increase in RMSD (Fig. 7*B*). The fluctuation of individual residues because of conformational changes is shown by root mean square fluctuation analysis that indicates an increased fluctuation at the C-terminal region resulting in peptide unfolding (Fig. 7*C*). SA-XV also exhibited a higher solvent-accessible surface area compared with SA-XII (Fig. S7*B*), which indicates the higher conformational changes in SA-XV when interacting with the membrane. The formation of an increased number of uninterrupted hydrogen bonds between SA-XV and the membrane was observed, as shown in Fig. S7*C*.

### SA-XV aids in wound healing of corneal cells

**Figure 8:**
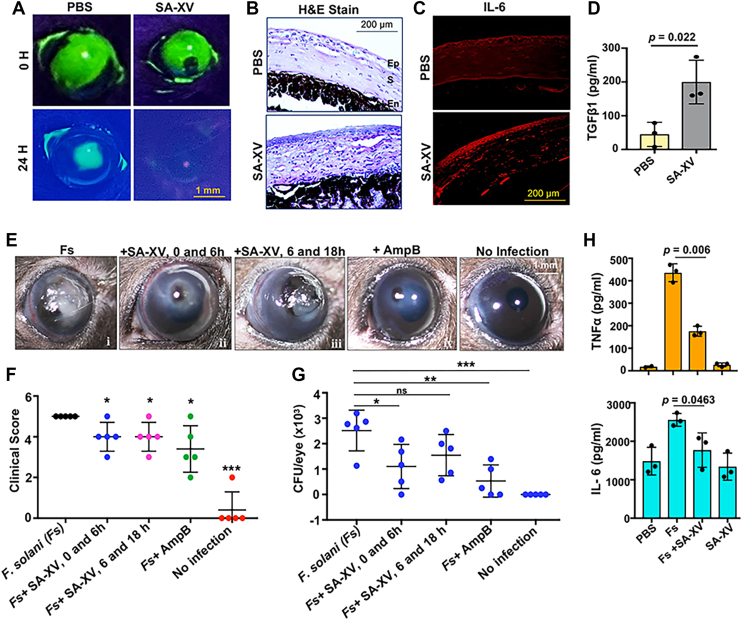
**SA-XV promotes corneal wound healing and inhibits fungal growth *in vivo*.** Representative images showing fluorescein-stained corneal wounds in C57BL/6 mice (n = 5) of PBS- or SA-XV-treated groups at 0 and 24 h (*A*). Corneal sections were obtained 24 h postwound from both control and treated enucleated eyes (n = 3 mice) and stained with haematoxylin and eosin to visualize the cellular infiltration and extent of wound present. En, endothelium; Ep, epithelium; S, stroma (*B*). Immunohistochemistry was done on murine corneal sections (n = 3) wounded and treated with or without SA-XV to determine the expression of interleukin 6 (IL-6) (*C*). Eyes (n = 3) were enucleated 24 h postwound, homogenized in PBS, and transforming growth factor beta 1 (TGF-β1) level was determined by ELISA (*D*). C57BL/6 mice (n = 5/group) were infected with *Fusarium solani* and topically treated with PBS or SA-XV at 0 and 6 h or SA-XV at 6 and 18 h postinfection. A group of mice infected and treated with amphotericin b (AmpB) was also included as a positive control. Mice were euthanized 48 h postinfection, and representative images of corneal opacification (*E*) and their clinical score (*F*) were recorded. Colony-forming unit (CFU) was measured from whole eye homogenate 48 h postinfection, and data points represent individual infected corneas (*G*). The level of tumor necrosis factor alpha (TNFα) and IL-6 was determined from eye homogenate (n = 3) infected and treated with or without SA-XV to determine its immunomodulatory role (*H*). (∗ denotes *p* < 0.05, ∗∗ denotes *p* < 0.005, ∗∗∗ denotes *p* < 0.0005, ns. denotes not significant).

We next sought to determine if SA-XV has any immunomodulatory effect on epithelial cells similar to the parent peptide S100A12 (16) and if it can accelerate wound healing. A scratch was made on the confluent layer of HCEC, and the migration of cells was documented every 2 h. The migration of cells to cover the wounded area is the first and an important event in the repair process (40). We observed closure of the wound completely by 20 h in SA-XV-treated cells compared with control untreated cells (Fig. S9*A*). The area of the closed wound was quantitated and graphically presented in Fig. S9*B*, which shows almost 20% more closure in peptide-treated cells. The epidermal growth factor receptor (EGFR) signaling pathway is known to play a pivotal role in the wound-healing process in keratocytes and corneal cells (41), and we observed increased phosphorylation of EGFR in HCECs by SA-XV as early as 30 min (Fig. S9c). Further, to check if SA-XV can aid in wound healing *in vivo*, corneal wounds were made by scratches in C57BL/6 mice (n = 5) (42) and treated topically with SA-XV (5 μg) at 0 and 6 h or saline for control eyes. The representative images of corneas at 0 and 24 h post wound are shown in Figure 8*A*. Improved wound healing was noted in corneas treated with SA-XV, as evident from reduced fluorescence, compared with PBS-treated corneas. The fluorescein-stained images of wounded eyes from all other mice are shown in Fig. S9*D*. The remaining wound area was quantitated by ImageJ software and represented graphically (Fig. S9*E*). The representative images of H&E-stained corneal sections of PBS and SA-XV-treated eyes are shown in Figure 8*B*, which clearly indicates increased cellular infiltrations in peptide-treated corneal sections. A similar observation of increased cellular migration was noted in wounded corneas treated with S100A12, the parent peptide (13). These infiltrating cells were mostly macrophages, as determined from F4/80 staining (Fig. S9*F*). The process of healing of corneal wounds is a dynamic process involving cell proliferation, differentiation, and tissue remodeling (43). We checked the expression of interleukin 6 (IL-6), as it has been known to facilitate wound healing of corneal cells both *in vitro* (44) and *in vivo* (45). We observed increased expression of IL-6 in corneas treated with SA-XV (Fig. 8*C*), indicating its role in mediating wound closure in this case. Increased expression of *IL-6*, tumor necrosis factor alpha (*TNFα*), and *EGFR* genes was also observed in the SA-XV-treated eyes by quantitative PCR (Fig. S9*G*). Transforming growth factor beta 1 (TGF-β1) is also known to stimulate corneal epithelial cell migration *via* activation of the mitogen-activated protein kinase pathway (46); a significantly increased level of TGF-β1 (Fig. 8*D*) was observed in eyes treated with SA-XV compared with control eyes. These data collectively indicate that SA-XV facilitates wound healing in corneal cells by increasing expression of several mediators and activating EGFR signaling pathways.

### SA-XV restricts fungal burden in a murine model of *Fusarium* keratitis

To determine if SA-XV could be clinically useful, we next tested the efficacy of the peptide using our well-established *in vivo* murine model of fungal keratitis (13). Infected mice were either treated with PBS (vehicle control, first group), with peptide SA-XV (5 μg) at 0 and 6 h postinfection (second group), or with peptide SA-XV (5 μg) at 6 and 18 h postinfection (third group). An additional group of infected mice was treated with AmpB at 0 and 6 h (fourth group). Mice infected with *F. solani* demonstrated an increased degree of corneal disease as reflected by enhanced corneal opacification and swelling and surface irregularity (Fig. 8*E*i) compared with reduced opacification in infected mice treated with SA-XV for either the second group (Fig. 8*E*ii) or the third group (Fig. 8*E*iii) at 48 h postinfection. Distinct reduced opacification was also noted in the infected group treated with AmpB, the currently used antifungal (Fig. 8*E*iv). No corneal opacification was noted in uninfected corneas (Fig. 8*E*v). The opacities were scored, and significant differences were noted in clinical scores of corneal opacities between the infected group and peptide-treated group (Fig. 8*F*), indicating a reduction in disease development. Consistent with these data, significantly reduced CFU was obtained from mice eyes treated with SA-XV at 0 and 6 h (*p* = 0.0284) postinfection, compared with *Fusarium*-infected but untreated corneas (Fig. 8*G*). Reduced CFU was also obtained from the group treated with SA-XV at 6 and 18 h postinfection compared with *Fusarium*-infected corneas; however, it was not statistically significant (*p* = 0.0934). A statistically significant reduction of CFU was also observed in the group treated with AmpB (*p* = 0.0025) compared with the *Fusarium*-infected group (Fig. 8*G*); however, there was no significant difference between this group and the peptide-treated group at 0 and 6 h postinfection (*p* = 0.2696). We also tested if SA-XV could modulate host immune responses during infections that could potentially benefit the management of corneal infections. Infected eyes with or without SA-XV treatment (n = 3) were homogenized, and cytokines were quantified by ELISA. While *Fusarium* infection significantly increased production of proinflammatory cytokines like TNFα and IL-6 compared with uninfected corneas, treatment with SA-XV was found to significantly reduce both TNFα and IL-6 secretion, implying its immunomodulatory role (Fig. 8*H*).

Next, the ADMET (Absorption, Distribution, Metabolism, Excretion, and Toxicity) properties of SA-XV were analyzed using the ADMET-AI server (https://admet.ai.greenstonebio.com) (47). Machine learning predictions indicated that the peptide possesses low human intestinal absorption, is clinically nontoxic, does not cross the blood–brain barrier, and has low human ether-a-go-go-related gene channel toxicity (Fig. S10*A*). The *in vivo* safety study of the peptide SA-XV was checked in a separate group in healthy mice eyes (n = 3). The peptide was added four times on the mice corneas at an interval of 3 h for 2 days and imaged. The fellow eye received PBS at the same time points. Although a small erosion was detected in one of the eyes treated with PBS (Fig. S10*B*ii), no corneal erosions or opacity were observed in the eyes treated with the peptide (Fig. S10*B*), and no edema or hyperemia was present. The histopathology images of corneal sections were analyzed, and no damages or abnormalities were noted, indicating the tolerability of the peptide (Fig. S10*C*). Taken together, these data suggest that the peptide SA-XV is biocompatible and possesses immunomodulatory properties along with its antifungal function.

## Discussion

The steep rise in resistance against current antifungals and increased mortality because of fungal infections (48) make it necessary to develop new therapeutics to combat fungal infections. We had reported regarding the antifungal properties of S100A12 against *Fusarium* spp. in the recent past (13); however, there are a few challenges in developing it into an ocular drug, the large size of the protein being one. In the current study, we determined a shorter 15mer fragment of S100A12, SA-XV, which retains the antifungal property of the parent peptide against both filamentous *Fusarium* spp. and nonfilamentous *Candida* spp. *in vitro* and in the murine keratitis model. We also elucidated the mode of action of this 15mer fragment with several lines of evidence against the fungal species and characterized its 3D structure. Furthermore, no apparent cytotoxicity was detected in HCECs when incubated with the peptide.

SA-XV not only exhibited antifungal activity against fungal species but also inhibited biofilm formation and significantly reduced preformed biofilms by *F. solani*. Interestingly, shortening the peptide further by three more amino acids at the C-terminal region completely abolished its antifungal property, indicating that the terminal three residues play an important role in inhibiting fungal growth. Further mutational analysis is underway to better understand the interaction of these amino acids with the fungal membrane. The environmental pH had an impact on the antifungal activity of SA-XV, and increased inhibition of fungal growth was noted at an acidic pH of 5.5 compared with that of pH 7.5. Earlier, there had been a report of crocodile defensin CpoBD13 that exhibited pH-sensitive action against *Candida* (31).

In an attempt to elucidate the mechanism of action of SA-XV, we found that the peptide directly binds with the mannan present in abundance in the cell wall of both *Fusarium* and *Candida* in 1:1 binding kinetics. Interestingly, we found that SA-XV does not bind to galactomannan present amply on the cell wall of *Aspergillus* spp. and failed to inhibit the growth of *A. flavus*. This indicates that binding of the peptide to the cell wall might be an important step for antifungal activity of SA-XV and that the specificity of the peptide toward *Fusarium* and *Candida* will prevent attainment of resistance among other species and minimize off-target effects. LL-37, another HDP, was also reported to bind to the cell wall polysaccharides of *Candida* and reduce adhesion of *Candida* to host cells (49). Present below the cell wall, phospholipids are the main constituents of the plasma membrane that influence cell growth and virulence (50) and play an important role in cell signaling (51). Similar to several plant defensins like NaD1 (52), MtDef5, and NCR044 (53), we found that SA-XV binds to several phospholipids like PIP2, PI3P, PI4P, and CL in a phospholipid-protein overlay assay. We had also reported earlier the binding of S100A12 to phospholipids, mainly phosphatidic acid and phosphatidylserine (13). Further studies are required to understand the role of phospholipid binding of SA-XV to induce membrane permeabilization. The removal of the cell wall does not influence permeabilization for peptides that directly bind to the plasma membrane (54). SA-XV was unable to permeabilize the plasma membrane when the outer proteinaceous layer was removed, which was also reported for NaD1 (55).

In contrast to S100A12, which was restricted to the cell surface (13), SA-XV enters fungal cytoplasm within 30 min in an energy-dependent mode and colocalizes within the nucleus and mitochondria. The translocation of SA-XV happens with a concomitant influx of PI, indicating plasma membrane perturbation caused by SA-XV. The mechanism involving plasma membrane permeation and targeting intracellular organelles has been reported earlier. Buforin 2 forms toroidal pores in the cell membrane and also binds to DNA and RNA (56); 14-helical β-peptides have also been shown to disrupt plasma, nuclear, and vacuolar membranes in *C. albicans* (57). Several AMPs are known to target mitochondria in different cellular systems and undermine the mitochondrial functions (58). The involvement of mitochondria in the killing process by SA-XV was demonstrated by colocalization of the peptide with the mitochondria-specific probe and binding to the CL present significantly in the IMM, causing membrane depolarization. Interestingly, CL plays a vital role in the membrane-perturbing activity of the rabbit neutrophil defensin (59). SA-XV also caused reduction in ATP generation. Because of the essential role of the mitochondria for fungal cell survival and virulence, targeting mitochondria by SA-XV might provide rich opportunities to develop it as a potent antifungal. SA-XV was also found to directly bind to the gDNA, effectively inhibiting the *in vitro* transcription process, and also caused cell cycle inhibition in both *Fusarium* and *Candida*. Numerous AMPs are known to bind to DNA and inhibit downstream transcription and translation (60). Several AMPs are known to induce ROS generation and cause apoptotic death in fungal pathogens (61). In this study, we found SA-XV induced ROS generation in *Fusarium* similar to S100A12, caused increased release of cytochrome *c*, and triggered DNA fragmentation as evident from TUNEL staining. We found that translocation of the peptide into the fungal cells causes formation of pores of greater than 2.8 nm within 2 h. The scanning electron microscope data also clearly illustrate the pore formation by 6 h and complete rupture of the membrane and cell damage in *Fusarium* and *Candida* by 24 h of incubation with peptide.

Three-dimensional structural elucidation using solution-state NMR showed the role of aromatic Y10 residue in mediating changes in the secondary structural conformations in the SDS micellar environment. The role of H9, H11, and H13 in electrostatic interactions with negatively charged SDS head groups may be responsible for binding to the membrane surface and exerting its mode of action. The peptide remains weakly bound to the membrane surface with the flexible C-terminal tail exposed to the solvent. The results of our MD simulations suggest that SA-XV tends to undertake a random coil conformation from its original helical structure in contact with the fungal membrane, which is also evident from the CD spectroscopy data. Molecular docking of SA-XV with DNA shows that there is a propensity of SA-XV binding to nucleic acids, with the positively charged residues driving the interaction between them. Whether they bind to a specific sequence motif is still unclear.

HDPs are often immunomodulatory in nature and have been reported to induce cell proliferation and aid in wound healing (62, 63). Similar to S100A12 (16), SA-XV also stimulates cell migration of corneal epithelial cells and activates EGFR pathways. SA-XV also accelerated wound healing in *in vivo* C57BL/6 corneas, along with increased expression of IL-6 and TGF-β1. Another endogenous HDP, histatin-5, has been shown to promote cell migration of corneal epithelial cells and helps in wound healing by activation of extracellular signal–regulated kinase pathways (64). We also demonstrated that, similar to the parent peptide S100A12, SA-XV is also effective in inhibiting *F. solani* corneal infections *in vivo* with significant reduction of corneal opacity and fungal burden in infected corneas treated with SA-XV compared to untreated ones. It also played an immunomodulatory role and reduced proinflammatory cytokine secretion in the *in vivo* model of corneal infection. Clark *et al.* (65) have earlier shown S100A8/S100A9 to be effective in regulating hyphal growth in *Aspergillus fumigatus* corneal infections. The immunomodulatory role of SA-XV was also determined in the presence of infection, and SA-XV suppressed inflammatory responses that are crucial in healing corneal infections and maintaining transparency. There is evidence of AMPs that efficiently tackled both infection and stimulated wound healing in corneal infection models. The peptides, Esc(1–21) and Esc(1–21)-1c, both derived from amphibian skin glands, have been shown to exhibit both antibacterial and wound-healing properties in bacterial keratitis (66). Similarly, keratin 6a-derived AMP effectively alleviated experimental bacterial keratitis and suppressed inflammation (67).

**Figure 9:**
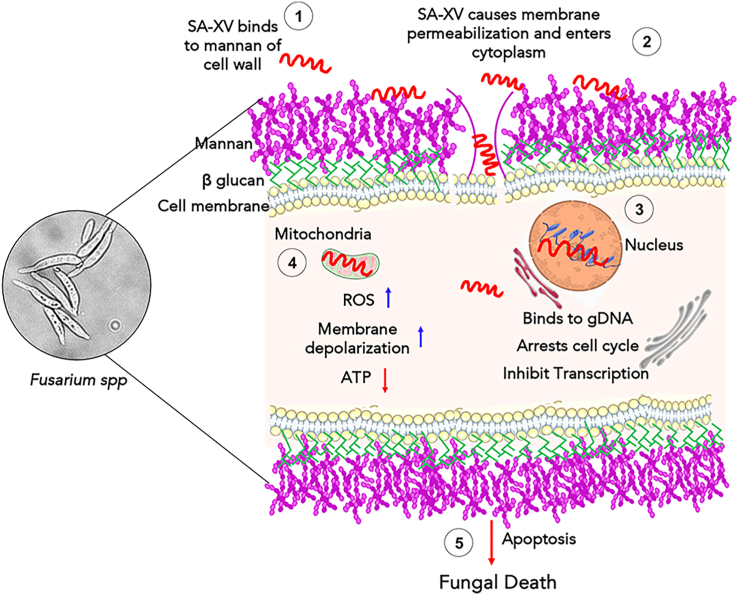
**Mode of action of SA-XV.** Schematic illustrations depicting the mechanism of action of SA-XV toward fungal cells leading to its death.

In summary, the multifaceted mechanisms of SA-XV have been revealed in this study that enable effective killing of both filamentous *Fusarium* and nonfilamentous *Candida* by the following sequence of events (Figure 9): (1) interaction of SA-XV with fungal cell wall and plasma membrane, (2) translocation across cell membrane and accumulation in the cytoplasm, (3) colocalization into the nucleus, binding to gDNA, and arresting the cell cycle, (4) targeting and permeabilization of mitochondria, and (5) causing fungal cell death by apoptosis. It also demonstrates dual functions, exhibiting both therapeutic potential against *F. solani*-induced corneal infections *in vivo* and wound-healing ability. Therefore, SA-XV represents a unique drug candidate with the potential to treat fungal infections and elicit corneal wound healing. Further research would help in optimization and formulation development of SA-XV as a possible alternative novel intervention.

## Experimental procedures

### Peptide synthesis and physiochemical property analysis/structure prediction

SA-XV peptide and FITC-labeled (N-terminal) SA-XV were synthesized by SP^2^TX and GL Biochem, respectively. SA-XII peptide was synthesized by GenScript Ltd. The purity of the peptides was confirmed to be higher than 97% by RP-HPLC and MALDI-TOF MS analysis. The peptides were reconstituted in sterile MiliQ water at a concentration of 5 mg/ml (3.1 mM). The predicted 3D structure of SA-XII and SA-XV was created using the online PEP-FOLD 4 structure prediction tool (22). The helical wheel projection was done by using the online HeliQuest tool (68).

### Antifungal assay

The ocular clinical isolates of *F. solani* (n = 5) and *C. albicans* (n = 5) were obtained from Jhaveri Microbiology Centre, LVPEI, India. The isolates were grown at 29 °C in Sabouraud dextrose broth (SDB; Himedia) for 48 h. *Fusarium* conidia (spores) were harvested as described before (69), centrifuged at 8000 rpm for 15 min to pellet the conidia, washed in 10 mM phosphate buffer (pH 7.4) twice, and resuspended in the same. *Candida* was grown in SDB overnight, centrifuged, washed, and resuspended similarly. *F. solani* and *C. albicans* (1 × 10^4^ conidia) were grown in the presence or absence of different concentrations of SA-XV in SDB in a 96-well microtiter plate and incubated at 29 °C for 24 h. Negative controls consisting of fungus only and a positive control consisting of fungus treated with AmpB (55 μM) were incubated simultaneously. The culture was then diluted accordingly and plated in potato dextrose agar (PDA) plates and incubated further for 24 h. The effect of the peptide was determined by quantifying the CFUs on the PDA plates. The effect of SA-XV on *A. flavus* was also determined similarly. All the experiments were performed at least thrice and in triplicate.

### Time-kill kinetics of SA-XV against *Fusarium*

Time-killing kinetics of SA-XV against *F. solani* was performed according to standard microbiological techniques with minor modifications (70). Freshly harvested *F. solani* (10^4^ conidia) were incubated with 60 μM of SA-XV at 29 °C for different time intervals (0, 2, 4, 8, and 24 h). To quantify the fungal population at different time intervals, cultures were taken, serially diluted in SDB, and plated on PDA plates, and incubated further at 29 °C for 24 h, and CFUs were quantitated. SDB medium was used as a negative control.

### Biofilm assay

The biofilm inhibition effect of SA-XV against *Fusarium* spp. was determined as described earlier (71). In brief, fungal spores (10^4^ conidia/200 μl media) were incubated with SA-XV (60 μM) for 24 h at 29 °C. In a separate experiment, the biofilm was first formed for 24 h followed by incubation with the peptide further for 24 h. The media supernatants were removed, wells were washed with 1× PBS, the biofilms were fixed using 95% methanol for 15 min, followed by staining with 0.5% of crystal violet (Sigma–Aldrich) for 15 min. The wells were further rinsed with distilled water, and 30% acetic acid was added to dissolve the dye, and absorbance was measured at 590 nm by the SpectraMax M3 Reader using Softmax Pro 6.3 software (Molecular Devices).

### ROS measurement

The intracellular ROS generation in *F. solani* was measured using 2′,7′-dichlorofluorescin diacetate (Invitrogen) as described before (72). *Fusarium* spp. (10^6^ conidia/ml) were incubated with 2′,7′-dichlorofluorescin diacetate in the presence or absence of SA-XV for 2 h, washed with 1× PBS, and observed under a fluorescent microscope (Olympus IX73) using 20× objective and imaged using an Olympus DP71 camera.

### Scanning electron microscopy

*Fusarium* spp. or *Candida* spp. (1 × 10^4^ conidia) were grown on the glass cover slips in the presence or absence of SA-XV (60 μM) for 6 h or 24 h, washed, and fixed with 4% formaldehyde (in 0.2 M cacodylic acid buffer). After fixation, the cover slips were washed again and dehydrated with graded ethanol (10%, 25%, 50%, 70%, 90%, and 100%) and air dried overnight. The cover slips were then sputtered with gold palladium for 75 s with a high vacuum evaporator and visualized using a scanning electron microscope (Carl Zeiss-Model EVO 18; Carl Zeiss) at a magnification of 5000×.

### Surface plasmon resonance

Binding affinity analysis for SA-XV with mannan and galactomannan (Sigma) was performed using SPR on a BIACORE T200 Instrument (GE Healthcare). The peptide was covalently coupled to a CM5 sensor chip using a standard 1-ethyl-3-(3-dimethylaminopropyl)carbodiimide/*N*-hydroxysuccinimide amine coupling method with sodium acetate buffer, pH 5.0. One flow cell was used as a reference and was immediately inactivated with 1 M ethanolamine, pH 8.5. In the other flow cells, mannan or galactomannan was injected at different concentrations with a flow rate of 30 μl/min. A blank channel (FC1) without SA-XV was used as a control for each experiment to correct for nonspecific binding and bulk changes in refractive index. The changes in mass because of the binding response were recorded as resonance units. The binding affinities of the peptides to mannan or galactomannan were measured using a 1:1 binding assay using Biacore T200 Evaluation Software 5.0 (GE Healthcare).

### Flow cytometry analysis of the cell cycle

*F. solani* or *C. albicans* were grown and diluted to 10^6^ cells/ml, as described earlier, and treated with SA-XV (60 μM) for different time points, whereas another set was kept untreated with the peptide. The cells were then harvested and processed for flow cytometry analysis. The fungal cells were fixed in 70% chilled ethanol for 1 h, washed, and then treated with RNase at a concentration of 100 μg/ml for 2 h at 37 °C. Following this, the cells were stained with PI for 30 min, washed, and analyzed by flow cytometry.

### *In vitro* transcription assay

*In vitro* transcription assay was done using the commercial kit Riboprobe System-T7 (Promega), according to the instructions for positive control samples but adding different concentrations of peptide to the reactions. As a negative control, the DNA template was omitted from the reaction mix. After 2 h of incubation at 37 °C, samples were run in a 1.2% agarose gel in TAE buffer and imaged using the Gel Doc System (Bio-Rad).

### DNA–peptide binding and competitive binding assay

gDNA was isolated from *Candida* according to the manufacturer’s protocol (Sigma) and quantified using a nanodrop (Thermo Scientific). gDNA (300 ng) was incubated with different amounts of SA-XV at 37 °C for 4 h in 1× TE buffer. The samples of the reaction mixtures were run in a 1% agarose gel in TAE buffer containing EtBr, separated by electrophoresis, and imaged using the Gel Doc System. In separate experiments, gDNA was incubated with EtBr or DAPI for 1 h, followed by incubation with different concentrations of SA-XV for 4 h at 37 °C. Fluorescence detection was done using an excitation wavelength of 535 nm and an emission wavelength range of 580 to 700 nm for EtBr and 358 nm excitation wavelength with an emission range of 400 to 600 nm for DAPI, with a sampling interval of 10 nm.

### Transmission electron microscope

*F. solani* cells with or without SA-XV treatment were fixed with 2.5% glutaraldehyde in 0.1 M cacodyl buffer (pH 7.2) and were refixed in a 1.5% potassium permanganate solution. The preparations were contrasted en bloc with 1% uranyl acetate and dehydrated with an ethanol series, supersaturated, and embedded in LR White resin (73). Ultrathin sections were made and observed using a Tecnai G2 T20 X-TWIN (FEI) transmission electron microscope.

### Membrane depolarization

*Fusarium* spp. (10^4^ conidia) were left untreated or incubated with SA-XV (60 μM) for different time points, washed with 1× PBS, and further incubated with 100 nM of 3,3′-dipropylthiadicarbocyanine iodide dye (Sigma–Aldrich) for 30 min in the dark followed by the addition of 100 mM KCl as described earlier (74). The membrane potential was determined by flow cytometry.

### PI uptake and PI staining assay

*F. solani* or *C albicans* (10^6^ conidia) was incubated in the absence or presence of SA-XV (60 μM) for 2 h. The spores were washed with 1× PBS and further incubated with PI (1 mg/ml) for 15 min. The uptake of PI was then analyzed by flow cytometry or imaged using the confocal microscope (LSM 880; Zeiss).

### Immunocytochemistry

*Fusarium* spp. or *Candida* spp. (1 × 10^4^ conidia) were incubated with 60 μM of FITC-SA-XV at 4 °C or 29 °C or different time points in SDB. The conidia were then washed three times with Milli-Q water and fixed using 4% paraformaldehyde. In some experiments, DAPI (Vectashield) or MitoTracker (Invitrogen) were used to stain specific cell organelles. In a different experiment, cytochrome *c* was detected in peptide-treated or untreated cells by incubating with anti–cytochrome *c* antibody (1:100 dilution; BioLegend), followed by Alexa Fluor 488 secondary antibody (1:1000 dilution; Thermo Fisher Scientific). In a separate experiment, the spores were incubated with SA-XV for 4 h in the presence or absence of FD (SR LifeScience Solution) of different molecular weights, washed with Milli-Q water, and fixed using 4% paraformaldehyde. The conidia were imaged using a 63× objective in a confocal microscope (LSM 880; Zeiss).

### ^1^H-coupled ^13^C NMR experiment and ^31^P solid-state NMR experiment

*Fusarium* spp. were grown in media containing yeast extract, peptone, and ^13^C-labeled glucose for 48 h under 120 rpm, harvested as per protocol, and washed thoroughly and resuspended in 10 mM phosphate buffer (pH 7.4). A total count of 10^7^ spores/ml was taken in an NMR tube. All NMR experiments were performed at 298 K on a Bruker Avance III 700 MHz NMR spectrometer. Ten percent of deuterated water and 3-(trimethylsilyl)-2,2,3,3-tetradeuteropropionic acid were used for locking and internal standard (0.00 ppm), respectively. First a free 1D ^1^H NMR spectrum of the free cell was recorded followed by a series of 1D ^1^H (water suppression using excitation sculpting) and ^1^H-coupled ^13^C-NMR (zggd30, 1D sequence with gated decoupling using 30° flip angle) spectra were recorded after addition of 0.5 mM SA-XV. Appearance of new peaks was observed in the 1D ^13^C because of the efflux of metabolites from the cells.

A single 90° pulse ^31^P solid-state NMR experiment was performed on an Agilent NMR spectrometer (DD2) equipped with a 4 mm MAS HXY Solid Probe. Resonance frequencies were 699.88 MHz and 283.31 MHz for ^1^H and ^31^P, respectively. The TPPM ^1^H-decoupled condition was used. LUVs were kept in a diameter of 4 mm Pyrex glass tube sealed with parafilm at an ambient temperature. ^31^P spectra were collected with 512∼1000 scans and a recycle delay of 2 s. The spectra were referenced externally to 85% phosphoric acid (0 ppm).

### Liposome preparation and calcein dye leakage assay

1-Palmitoyl-2-oleoyl-*sn*-3-glycero-phosphoethanolamine (POPE) and 1-palmitoyl-2-oleoyl-*sn*-3-glycero-phosphatidylcholine (POPC) were purchased from Avanti Polar Lipids Inc. CL, CHL, and ERG were purchased from Sigma–Aldrich Co. Lipid stock solutions were prepared in chloroform at a concentration of 25 mg/ml. Fungal model membrane mimics composing 5:4:3 POPC/POPE/ERG were created following the standard protocol. Liposomes mimicking the IMM composed of POPC/POPE/CL/CHL were also prepared using the standard protocol. The lipid film was created after drying in a stream of nitrogen gas and lyophilizing overnight. The lipid film was then hydrated with 70 mM calcein dissolved in 10 mM Tris buffer (pH 7.4), followed by vigorous vortexing for 30 min and subjecting to five freeze–thaw cycles in liquid nitrogen and lukewarm water to obtain the dye-entrapped vesicles. The vesicles were then passed through a 100-nm polycarbonate membrane filter stacked in a mini extruder (Avanti Polar Lipids) set up to obtain LUVs. The free calcein dye was removed by passing the extruded samples through a gel filtration–based hydrated Centrisep-Spin Column (Thermo Fisher Scientific), which was centrifuged at 3000 rpm for 2 min.

Dye-encapsulated liposomes were suspended in an extravesicular buffer containing 10 mM Tris buffer and 100 mM NaCl (pH 7.4). The leakage of calcein caused by liposome disruption was analyzed by fluorescence emission at 519 nm using a JASCO F-8500 fluorescence spectrophotometer. The slit width for both excitation and emission was kept at 5 nm. S100A12 was added at functional concentrations, and enhancement in fluorescence was measured. Triton X-100 (0.1%) served as a positive control, completely disrupting the calcein-encapsulated unilamellar vesicles. Percentage leakage was calculated using the equation.

Dye leakage (%) = $\frac{\left(F-F_{0}\right)}{\left(F_{T}-F_{0}\right)}\times 100$%

where, *F*_*0*_, *F*, and *F*_*T*_ denote the basal fluorescence intensity, fluorescence intensity after addition of peptides, and maximum fluorescence intensity obtained after addition of 0.1% Triton X-100, respectively.

### Isothermal titration calorimetry

The thermodynamics of the interactions of SA-XV with *Fusarium* spp. or NDs was determined using TAaffinity isothermal titration calorimetry (TA Instruments). The peptide, NDs, and *Fusarium* cells were dissolved in 10 mM phosphate buffer (pH 4.5), filtered, and degassed. *Fusarium* or NDs were loaded in the sample cell to a volume of 182 μl and was titrated with 200 μM of SA-XV; loaded in the syringe. A total of 35 injections were performed in the case of *Fusarium*, whereas a total of 25 injections were performed in the case of NDs, with each injection containing 2 μl of peptide at an interval of 2 min, at 25 °C. Analysis of raw data was done using 3.7.5 software provided with the instrument. Fitting of each plot was done employing an independent binding site model to determine the number of binding sites (n), dissociation constant (*K*_*D*_), change in enthalpy (ΔH), free energy of binding (ΔG), and entropy (ΔS). Gibbs free energy for both reactions was evaluated using the equations: ΔG = -RT ln KA and ΔG = ΔH - TΔS.

### Tyrosine fluorescence spectroscopy

The intrinsic fluorescence of tyrosine of SA-XV (Y10) in 10 mM phosphate buffer (pH 7.4) in the absence and presence of NDs was recorded using Hitachi F-7000 FL spectrophotometer. ND and SA-XV peptide were prepared in 10 mM phosphate buffer (pH 7.4). The fluorescence spectra of 10 μM peptide were measured upon increasing concentration of NDs at a spectral range of 300 to 400 nm using excitation wavelength of 280 nm, slit width of 5 nm, in a quartz cuvette of path length 0.1 cm.

### Dynamic light scattering

Dynamic light scattering experiments were performed in a Malvern Zetasizer Nano S (Malvern Instruments) equipped with a 4 mW He–Ne laser (633 nm) and a back-scattering angle of 173°. NDs and SA-XV were dissolved in 10 mM phosphate buffer (pH 7.4), filtered, and degassed. NDs were added with increasing concentrations of the peptide solution in low-volume disposable cuvettes. Autocorrelation data generated the particle size distributions from the non-negative least squares fit algorithm provided in the instrument (75).

### Quantitation of intracellular ATP level

Intracellular ATP levels of *Fusarium* spp. treated with different concentrations of SA-XV were quantified using the Bac Titer Glo Microbial Cell Viability Assay (Promega). Fusarium not exposed to peptide was used as a control. The plates were incubated at 37 °C for 15 h. Subsequently, Bac Titer-Glo reagent was added to each well and incubated further for 15 min. The luminescence of each plate was measured using the Varioskan LUX Multimode Microplate Reader (Thermo Fisher Scientific).

### ^31^P solid-state NMR

^31^P solid-state NMR experiments were performed using the Agilent NMR spectrometer (DD2) functioning at the resonance frequency of 283.31 MHz for ^31^P and 699.88 MHz for ^1^H, equipped with a 4 mm MAS HXY Solid Probe. MLVs were prepared by dissolving POPC, POPE, and ERG (5:4:3) in chloroform to obtain a stock of 5 mg/ml. The lipid mixtures were dried under nitrogen gas, lyophilized overnight followed by dissolving the lipid film in Tris buffer (pH 7.4), and hydrated for 1 h by vortexing occasionally and freeze–thawing five times. A single 90◦ pulse and 24 kHz TPPM proton decoupling were used. The π/2 pulse lengths were set at 6.8 μs for the ^31^P nucleus. MLVs were kept in a 4 mm Pyrex glass tube, which was fit into the MAS probe, and sealed with parafilm. The sample temperature was maintained at 25 °C using an Agilent temperature control unit. ^31^P spectra were collected with 256 scans and have a cycle delay of 2 s and are referenced externally to 85% phosphoric acid (0 ppm). MestReNova software (version 8.1) with 250 Hz line broadening was used for spectra processing.

### Solution-state NMR

NMR spectrometer (700 MHz) equipped with a 5 mm RT probe was used for NMR experiments. Briefly, NMR samples were prepared in 10% deuterated buffer (pH 4.5), and 3-(trimethylsilyl)propionic-2,2,3,3-d4 acid sodium salt was used as an internal standard. Two-dimensional ^1^H–^1^H TOCSY and 2D ^1^H–^1^H 2D NOESY were recorded for the SA-XV peptide. A spectral width of 12 ppm was set in both directions. The number of scans was fixed to 20 and 160 for TOCSY and NOESY, respectively. The recycle delay (D1) for both the experiments was set to 1.5 s with 456 increments in the t1 and 2048 data points in the t2 dimensions along with state-time-proportional phase incrementation for quadrature detection in the t1 dimension, and an excitation-sculpting scheme for water suppression was used to record both the experiments (76). SDS (200 mM) was added to the peptide solution, and the change in spectra was monitored by ^1^H NMR acquired using an excitation-sculpting scheme for water suppression and the states-time-proportional phase incrementation for quadrature detection in the t1 dimension (77). 2D TOCSY and NOESY spectra of the peptide in the presence of SDS micelles were acquired with 80 ms and 150 ms mixing time, respectively. Topspin v3.1 software (Bruker Biospin) and Sparky [26] software were used for data acquisition and analysis.

### NMR-derived structure calculation

The 3D structure of SDS micelle–bound peptide was calculated. Depending on the intensities in the NOESY spectra, the volume integrals of the respective NOE crosspeaks were qualitatively differentiated into strong, medium, and weak with interproton upper bound distances of ≤3.0, 3.5 to 4.0, and 4.0 to 5.0 Å, respectively, whereas the lower bound distance was fixed to 2.0 Å. For all nonglycine residues, the backbone dihedral angles of the peptides, phi (ϕ) and psi (ψ), were kept flexible (-30◦ to 120◦ and 120◦ to -120◦, respectively) to limit the conformational space. All structure calculations were done using CYANA program, version 2.1 (78), and iterative refinement of the structure based on distance violation was performed. All hydrogen bonding constraints were eliminated from structure calculation. The NMR-derived ensemble structures were analyzed using PyMOL software, and their stereochemistry was checked using PROCHECK (79).

### All-atom MD

All-atom MD simulations were done to understand the interaction of two peptides, SA-XV and SA-XII, in a horizontal orientation with the fungal membrane using GROMACS 2023.3 (80). The modified fungal bilayer membrane (without glycosyl inositol phospho ceramide) was built with the CHARMM-GUI (81, 82) program using the CHARMM36 force field for lipids (83, 84). Bilayers were designed symmetrically, with an equal number of lipids in each leaflet comprising 66 counts of ERG and 14 counts each of DPPC, DPPE, DPPG, DUPC, SLPE, and SLPG. The peptides were placed 25 Å above the fungal membrane from the center, with a salt concentration of 150 mM NaCl using CHARMM-GUI. The entire setup was energy minimized for 5000 steps and equilibrated with constant volume twice for 1 ns and constant pressure four times for 1 ns. The production run was setup for 100 ns. MD simulations were carried out with CHARMM36 force field (83) and TIP3P water model (85). Particle mesh Ewald was used to compute the system's electrostatic interactions, and a cutoff of 12 Å was used for van der Waals interactions. The LINCS (Linear Constraint Solver) algorithm was employed to manage hydrogen bond constraints during the MD simulations (86). The MD simulations were performed with an integration time step of 2 fs, and the neighbor lists were updated every 20 steps. All groups used velocity rescale temperature coupling with a time constant of 1.0 ps. The atmospheric pressure was maintained at 1 bar using weak semi-isotropic pressure coupling with Kz = Kxy = 4.5 × 10^-5^ bar^-1^ and a time constant τ_P_ = 5.0 ps.

### Murine model of corneal wound healing

The murine model of corneal wound was developed as described earlier (87) with some modifications. Briefly, C57BL/6 mice (6–8 weeks old; n = 5) were anesthetized, and center corneas were carefully marked with 1.5 mm trephine and wounded using 0.5 mm burr of Algerbrush II (Alger Company) for 40 to 50 s. The right eyes received the SA-XV, whereas the left eyes received 1× PBS immediately after the procedure and 6 h postwound and were enucleated and processed at 24 h post-treatment for ELISA or histopathology. For determination of wound healing, corneas were stained with fluorescein (FLUO-fluorescein sodium ophthalmic strips; Care Group) 24 h post treatment. The eyes were viewed under a slit lamp microscope and imaged using a point-and-shoot camera, followed by enucleation. The fluorescent intensity of the dye was analyzed and quantified by ImageJ software (88). For histopathology, enucleated eyes (n = 3) were fixed in 10% formalin in PBS, 5 μm paraffin-embedded corneal sections were prepared, and hematoxylin and eosin staining was done as described before (89). Tissue sections were also deparaffinized and stained with mouse anti-IL6 and anti-F480 (eBioscience). All the studies that involved animals were approved by the Institutional Animal Ethics Committee (EAF/SR/03/2025) of Centre for DNA Fingerprinting and Diagnostics, Hyderabad, and carried out in accordance with the guidelines provided by the Association for Research in Vision and Ophthalmology (ARVO) for the use of animals in vision research.

### Murine model of *F. solani* keratitis

C57BL/6 mice (6–8 weeks old) were anesthetized by intraperitoneal injection using ketamine (8.7 mg/ml) and xylazine (0.5 mg/ml) at a dose of 120 μl/g body weight. While anesthetized, the corneal epithelium was abraded with three parallel vertical and horizontal scratches with a 26-gauge needle, and *F. solani* (10^7^ conidia in 2.5 μl) were added in one eye, whereas the fellow eye received 1× PBS (n = 5). In another anesthetized group (n = 5), mice were infected with *F. solani* followed by topical application of SA-XV (5 μg) to the corneas at 0 and 6 h postinfection. Another group of anesthetized mice (n = 5) was infected with *F. solani* followed by topical application of SA-XV (5 μg) to the corneas at 6 and 18 h postinfection. One more group of mice (n = 5) was infected with *F. solani* followed by topical application of AmpB (5 μg) at 0 and 6 h postinfection. Mice were euthanized and examined under a stereomicroscope for density and area of corneal opacification or ulceration 48 h postinfection and photographed using a digital camera. Clinical scores for the opacity were determined in a blinded fashion according to the scale earlier reported (90), eyes were enucleated and homogenized using a tissue homogenizer (Ez-Lyzer; Genetix Biotech) in sterile 1× PBS, and homogenates were plated on PDA plates for determining CFUs. All animals were housed in specific pathogen-free conditions in microisolator cages and were treated in accordance with the guidelines provided in the ARVO statement for the Use of Animals in Ophthalmic and Vision Research. The study was approved by the Institutional Animal Ethics Committee (EAF/SR/15/2023), Centre for DNA Fingerprinting and Diagnostics, India.

### Statistical analysis

Bar graphs represent the mean, and the error bars represent SD. All data were statistically analyzed; for comparisons between control and treated conditions, one-tailed, unpaired *t* tests were performed. Mann–Whitney test was used for animal experiments. All statistical analysis and graphics building were done using Prism7 (GraphPad Software). Results were considered statistically significant when *p* < 0.05.

## Data availability

All data needed to evaluate the conclusions are present in the article and/or the supporting information.

## Supporting information

This article contains supporting information (14, 16, 88, 91, 92, 93, 94, 95, 96, 97).

## Conflict of interest

D.H.S. is part of the SP^2^TX company but has no role in experimental design or interpretation of data. The authors declare that they have no conflicts of interests with the content of this article.
