# Supplementary material for: SA-XV, a 15-amino acid fragment of host defense peptide S100A12, targets mitochondria and is protective against fungal infections
Source: J Biol Chem. 2025 Sep 18;301(10):110743. doi: 10.1016/j.jbc.2025.110743 (PMC12552953; doi:10.1016/j.jbc.2025.110743)
Supplement: Supporting Information [file mmc1.pdf]

SA-XV, a 15-amino acid fragment of host defense peptide S100A12, targets mitochondria, and is protective against fungal infections

Riddhi Agarwal[1,2,3,#], Karishma Biswas[1,2,4,#], Akshita Agrawal[1,2,#], Nisha Nandhini Shankar[5], Srijita Kundu[1,2,3], Dipanwita Roy[1,2,4], DeokHyun Son[6], Amaravadhi Harikishore[8], Ragothaman M. Yennamalli[5], DongKuk Lee[6], Anirban Bhunia[4,*], Sanhita Roy,[1,2,*]

1. *Prof. Brien Holden Eye Research Centre, LV Prasad Eye Institute, Hyderabad 500034, India*
2. *Dr. Chigurupati Nageswara Rao Ocular Pharmacology Research Centre, LV Prasad Eye Institute,Hyderabad, India*
3. *Graduate Studies, Manipal Academy of Higher Education, Manipal, India*
4. *Department of Chemical Sciences, Bose Institute, Unified Academic Campus, Sector V, EN 80, Kolkata 700091, India*
5. *Department of Bioinformatics, School of Chemical and Biotechnology, SASTRA Deemed to be University, Thanjavur, India*
6. *Department of Fine Chemistry, Seoul National University of Science and Technology, Seoul 01811, Korea*
7. *SP^2 Therapeutics Inc (SP^{2}TX), 105-312 Startup Center of Inst. of Molecular Biology & Genetics, Seoul National University. Seoul, Korea*
8. *School of Biological Sciences, Nanyang Technological University, 60 Nanyang Drive, Singapore, Singapore 637551*

# Equal contributions

[*]Corresponding authors

Sanhita Roy, PhD

Prof. Brien Holden Eye Research Centre,
LV Prasad Eye Institute
Hyderabad-500034, India
Email: sanhita@lvpei.org
Telephone: +91-40-30612529; Fax: +91-40-30612535

Anirban Bhunia, PhD

Department of Chemical Sciences,
Bose Institute,
Sector V, EN 80,
Kolkata 700091, India
Email: bhunia@jcbose.ac.in
Telephone: +91-33-2569-3291

Running Title: *SA-XV inhibits fungal growth.*

Supporting Information

Supplementary experimental procedures

Cell culture and in vitro scratch assay

Immortalized human corneal epithelial cells (HCEC) 10.014 pRSV-T was maintained in DMEM-F12 media (Lonza, Walkersville, MD) as described earlier (1). Confluent monolayers of HCEC were scratched with a 200 µl sterile plastic pipette tip to create a wound. Cells were cultured further in media with presence or absence of SA-XV (6 µM) and followed for every 2 h until the closure of wound was observed using brightfield microscope with camera (Olympus A370, Zeiss, Germany). The wound area was further analyzed using an image analyzing software, Image J (2). The wound area was calculated as a percentage of the initial wound area.

Cytotoxicity assay

Cytotoxicity assays in human corneal epithelial cells (HCEC), sino-nasal epithelial cells (SNEC) or peripheral blood mononuclear cells (PBMC) were performed using MTT (Sigma-Aldrich, St. Louis, MO) as described earlier (3). In brief, cells ($2x10^4$ cells/well) were seeded into 96 well plates and cultured overnight. Cells were treated with different concentration of SA-XV for 24 h and cell viability was assessed by incubating the cells with 100 µL of MTT (2mg/mL) for 3 h. Formazan formation was quantified by dissolution of the crystals in dimethyl sulfoxide (DMSO, Sigma SRL, India), and absorbance was recorded at 570 nm by multiplate reader SpectraMax M3 (Softmax Pro 6.3). Viability was calculated as follows: Cell viability (%) = absorbance value of experiment well/absorbance value of control well x 100.

Hemolysis assay

Hemolytic assay was done as described before (4). In brief, erythrocytes were obtained from human blood and washed with 1X PBS three times. $1x10^7$ cells were treated with SA-XV (60 µM) for 2 h, centrifuged at 2500 rpm for 5 min and the absorbance of the supernatant was measured at 405 nm using a plate reader (Thermo Scientific,Waltham, MA). Cells incubated with media or 10% Triton X-100 were considered as negative and positive control respectively.

Prediction of stability, irritancy and safety assessment of the peptide SA-XV

The stability of SA-XV in the presence of Fetal Bovine Serum (FBS) was monitored as mentioned previously (5). Briefly, 1mg/ml of SA-XV was incubated in RPMI media supplemented with 25% FBS at 37˚C for 15 min. 100 µl aliquots were taken at different time intervals (0, 30, 60, 120, 180, and 240 min). 200 µl of 96% ethanol was added to precipitate the serum followed by centrifugation at 12000 x *g* for 2 min. 20 µl of the supernatant was subjected to a LC-20AT reverse phase HPLC system (SHIMADZU, Japan) equipped with Phenomenix C_{18} column (250x10 mm, pore size 100 Å, particle size 5 µm), at room temperature, using acetonitrile and water with 0.1 % TFA as the solvents. A flow rate of 1ml/min was maintained. The percentage of peptide remaining in the supernatant at different time intervals were analysed by peak integration using SPINCHROME CFR software, and the area obtained was normalized with respect to the free peptide.

The Hen's egg chorioallantoic membrane (HET-CAM) test was carried out to evaluate the irritancy of SA-XV as described before (6). SA-XV was added directly onto the chorioallantoic membrane surface containing vasculature by keeping the egg in the equator position for 5 min. The negative control was 0.9% w/v sodium chloride solution, and the positive control was 0.1 M sodium hydroxide solution. The membrane was crucially observed for any damage in blood

vessels, e.g., haemorrhage, clotting, or coagulation. The images were captured using a digital camera.

The safety assessment of the peptide was done by determining the ADMET properties of SA-XV using the ADMET-AI server (https://admet.ai.greenstonebio.com) (7). Further, selectivity index (SI) was determined from the ratio of the peptide concentrations that caused 50% cell cytotoxicity of healthy mammalian cells (CC_{50}) to the minimum inhibitory concentration (MIC_{90}), concentration at which 90% fungal death occurs. The formula used was $SI = CC_{50} / MIC_{90}$ 25 µg/ml

Competition assay

For the competition assays with mannan, SA-XV (60 µM) was preincubated with increasing concentration of Mannan (Sigma- Aldrich, St. Louis, MO) for 2 h at 4°C, followed by incubation with *Candida spp.* (10^4 spores) or *Fusarium spp.* (10^4 spores) for 6 h at 37°C or 29°C respectively. Post-incubation, the culture was serially diluted and plated on PDA plates, growth was quantitated by counting the colony forming units. Mannan stock solution was prepared in nuclease free water. Fungal cultures with no peptide treatment were used as a control.

Phospholipid binding assay

SA-XV-phospholipid interactions were determined using PIP Strips (Echelon Biosciences, Salt Lake City, UT) as described previously (13) with minor modifications. The binding of SA-XV (60 µM) to the phospholipids on strip was detected by anti-S100A12 antibody (1:100, Novus Biologicals, CO) followed by incubation with IRDye-680 secondary antibody (1:6000 dilution; LI-COR Biotechnology, Lincoln, NE) and were developed on Odyssey CLx Imaging System (LI-COR Biotechnology, NE).

Histology and Immunohistochemistry

Eyes form PBS and SA-XV treated mice were enucleated and placed in 10% formalin and tissue sections (5 µm) of paraffin-embedded corneas were obtained. The tissue sections were deparaffinised and hematoxylin and eosin (H&E) was done. Sections were also stained with anti-IL6 and anti-F480 antibodies (1:100; eBioscience) as described earlier (8), counterstained with DAPI (Vector Laboratories Inc, Newark, CA) and observed under fluorescent microscope (Olympus IX73, Zeiss, Germany) using 20X objective and imaged using Olympus DP71 camera.

RNA extraction, cDNA synthesis and qPCR

Enucleated murine eyes were homogenized and RNA was isolated using RNeasy Mini Kit (Qiagen, Hilden, Germany). cDNA synthesis was done using Verso cDNA synthesis kit (Thermo Scientific, Waltham, MA) according to the manufacturer's protocol. Quantitative PCR was performed on ABI PRISM 7000HT Sequence Detection System (Applied Biosystems, Grand Island, NY) using the SYBR Green PCR Master Mix (Thermo Fisher, Waltham, MA). Relative quantities of mRNA expression of respective genes were normalized using the $2^{-\Delta\Delta Ct}$ method using GAPDH as housekeeping gene. The primer sequences are shown in Table S2.

Preparation and characterization of nanodisc

i) Purification of MSP protein

In this study, we utilized the MSPD1ΔH**5** variant, as a scaffold protein to wrap nanodiscs, comprising 167 amino acids with a molecular weight of approximately 19.45 kDa. Assembly

with MSPD1ΔH5 typically yields nanodiscs with a diameter of ~9 nm. MSP expression and purification were performed in *E. coli* BL21 (DE3) cells following previously established protocols (9).

ii) Assembly setup of MSP protein and lipids to prepare nanodisc

Nanodiscs were mixed in the proportion mentioned in the table:

Lipid Mixture	MSP Variant	MSP/Lipid Ratio	Temperature applied during nanodisc assembly (°C)
41.6% POPC<br>33.3% POPE<br>25% Ergosterol	MSPD1ΔH5	1: 50	30

To enhance the aqueous solubility of ergosterol, a succinate group was conjugated, producing succinyl-ergosterol, which was incorporated into the nanodisc formulation. Accurate amounts of POPC, POPE, and succinyl-ergosterol were weighed and dissolved in an assembly buffer (20 mM Tris-HCl, pH 7.5, 100 mM NaCl, and 0.5 mM EDTA) supplemented with 20 mM sodium cholate. The MSP protein stock (500 µM) was prepared in the same buffer system. MSP was added dropwise to the lipid mixture under continuous stirring at approximately 180 rpm. The assembly mixture was incubated for 1 h at room temperature. Subsequently, detergent removal was facilitated by overnight incubation with Bio-Beads SM-2 (Bio-Rad, USA) at room temperature. Nanodisc-containing samples were recovered by centrifugation (1000 × g, 1 min) and subjected to further purification by size-exclusion chromatography.

Size-Exclusion Chromatography (SEC)

Following detergent removal, the nanodisc samples were immediately loaded onto a Superdex 200 Increase 10/300 GL column (GE Healthcare, Freiburg, Germany) pre-equilibrated with the assembly buffer. Purification was carried out using an ÄKTA FPLC system (GE Healthcare) at a flow rate of 0.4 mL/min. UV absorbance was monitored at 280 nm and 215 nm to track protein and lipid components, respectively. Fractions corresponding to nanodisc elution were collected and concentrated using centrifugal concentrators with a 10 kDa molecular weight cut-off (MWCO).

Transmission Electron Microscopy (TEM) of nanodiscs

Nanodiscs were visualized by transmission electron microscopy. Samples were diluted to 10 µM in Milli-Q water, and 10 µL was applied to carbon-coated copper grids (Electron Microscopy Sciences, USA). After a 2-minute incubation, excess sample was removed by blotting. Grids were subsequently washed with Milli-Q water and negatively stained with freshly prepared, filtered 1% (w/v) uranyl acetate solution for 5 minutes. The grids were air-dried overnight in a dust-free environment. TEM imaging was performed using a FEI Tecnai TF20 transmission electron microscope operating at 200 kV, providing a point resolution of 0.24 nm and a line resolution of 0.102 nm. Images were recorded digitally, and further analysis was conducted in energy-dispersive X-ray spectroscopy (EDS) mode.

Molecular Docking setup

The docking study was carried out using AutoDock 4.2.6 (10). The crystal structure of the DNA with the PDB Id: 4EFJ was downloaded from Protein Data Bank. The DNA file was prepared by removing water and extra chains. Polar hydrogen atoms and Kollman charges were added

to the DNA and to SA-XV peptide. A user-specified grid box of 88 × 126 × 126 was created with a grid point spacing of 0.686 Å and a grid center of -18.582, 33.141, and -16.150 was used to calculate the grid. SA-XV peptide was docked with the DNA using the lamarkian genetic algorithm (GA) in an exhaustive mode and 100 GA runs were setup. The top 100 clustered docking results were ranked in the increasing order of their binding energies. The peptide conformation analyzed using PyMOL and LigPlot+ (11).

Table S1: Effect of SA-XV against ocular clinical isolates

Fusarium spp. **Clinical Isolates**	**% Growth Inhibition by SA-XV (60 µM)**	***Candida spp.*** **Clinical Isolates**	**% Growth Inhibition by SA-XV (60 µM)**
Fusarium spp. 514/21	99.99	*C. albicans* 160/20	99.99
Fusarium spp. 213/21	99.99	*C. albicans* 3049/24	99.99
Fusarium spp. 217/21	99.99	*C. albicans* 4059/24	99.99
Fusarium spp. 91/21	99.99	*C. albicans* 259/24	99.99
Fusarium spp. 2644/20	99.99	*C. albicans* 5191/24	99.99

Table S2: Oligonucleotide Sequences

**Gene**	**Sequence (5'→3')**
EGFR	FWD:GGACTGTGTCTCCTGCCAGAAT REV:GGCAGACATTCTGGATGGCACT
IL-6	FWD:CTGCAAGAGACTTCCATCCAG REV:AGTGGTATAGACAGGTCTGTTGG
TNFα	FWD:GGTGCCTATGTCTCAGCCTCTT REV:GCCATAGAACTGATGAGAGGGAG
GAPDH	FWD:AGGTCGGTGTGAACGGATTTG REV:GTAGACCATGTAGTTGAGGTCA

Table S3: Structural statistics for the 20 lowest energy ensemble structures of SA-XV in presence of SDS micelle

	**SA-XV in presence of SDS micelle**
**Distance restraints**	
Intra residue (i - j = 0)	41
Sequential (|i - j| = 1)	33
Medium range (2 ≤ |i - j| ≤ 4)	11
Long range (|i - j| ≥ 5)	3
Total	**88**
**Angular restraints**	
Φ	14
Ψ	13
**Distance restraints from violation** (≥ 0.4 Å)	1
**Deviation from mean structure (Å)**	
Average backbone to mean structure	1.66 ± 0.55 Å
Average heavy atom to mean structure	2.11 ± 0.54 Å
**% Residues in Ramachandran plot***	
most favoured region	69.2
additionally allowed region	30.8
generously allowed region	0.0
disallowed region	0.0

Table S4: Receptor interface residues involved in hydrogen bonding, bond length and hydrophobic interactions.

Binding system	Receptor Interface residues involved (C and D represent the two strands of DNA)	Bond length (Å)
SA-XV -DNA	Hydrogen Bond	
	SAXV:VAL1:N - D:DG6:OP2	2.54
	SAXV:LYS14:NZ – C:DG17:OP1	2.70
	SAXV:TYR10:OH – C: DG17:OP2	3.12
	SAXV:HIS9:NE2 - C:DT19:OP2	3.31
	Hydrophobic interaction	-
	D:DT5	-
	D:DT7	-
	C:DT16	-
	C:DG18	-
DA: Adenine; DC: Cytosine; DG: Guanine; DT: Thymine		

Supporting Results

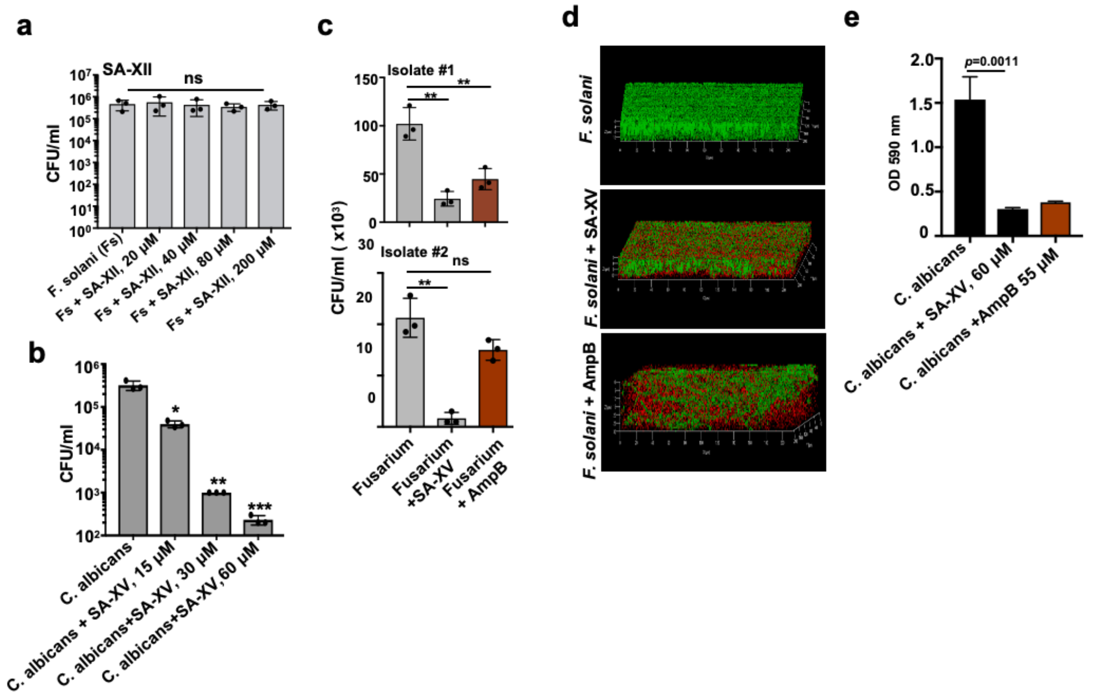

Supplementary Figure 1. Effect of peptides on different fungal species. Growth inhibition assay of SA-XII at different concentration against *Fusarium spp.* (a). The inhibitory effect of SA-XV at different concentration on *C. albicans* (b) or amphotericin resistant *Fusarium* clinical isolates (c) as determined by colony forming units (cfu). Confocal images of biofilm formed by *Fusarium spp.* in presence of SA-XV and amphotericin B stained with SYTO9 (green) and propidium iodide (red) (d). SA-XV inhibits biofilm formation of *C. albicans* as determined by crystal violet assay (e). (* denotes $p<0.05$, ** denotes $p<0.005$, *** denotes $p<0.0005$, ns denotes not significant)

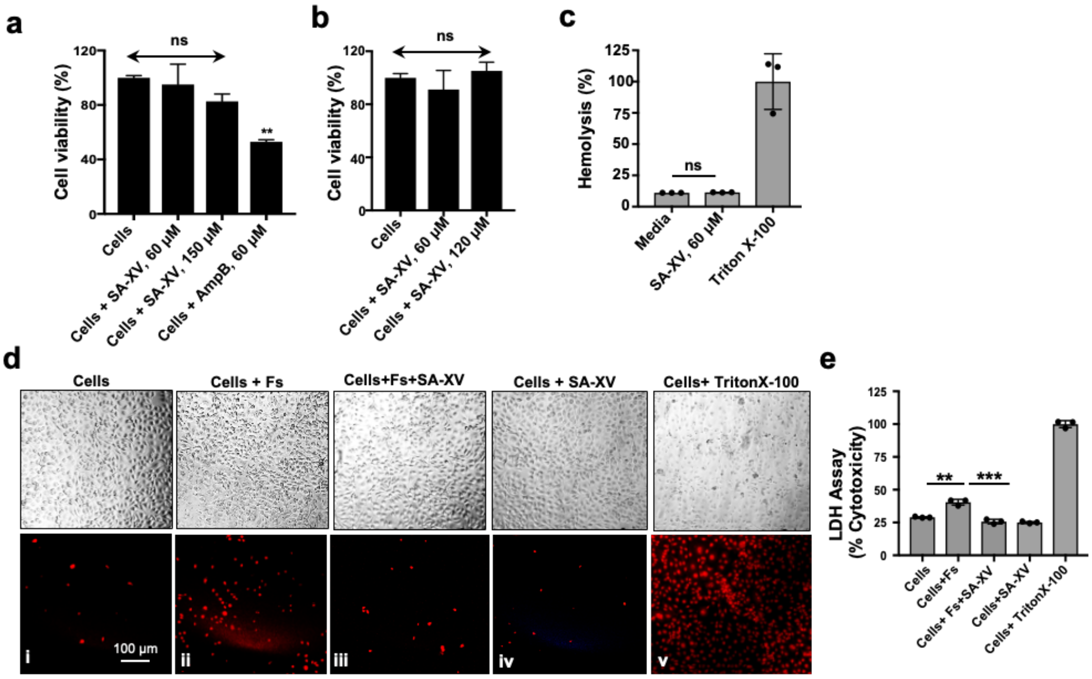

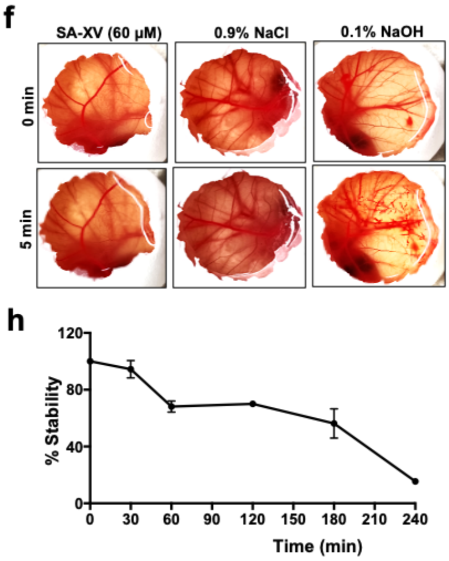

g

Conditions	Retention Time (min)
Stored in -30°C	8.30
Stored at 37°C (1 week)	8.29
Stored at 4°C (1 week)	8.30
Stored at -80°C (1 week)	8.30
Stored at -30°C (Freeze and thawed daily for 1 week)	8.30

Supplementary Figure 2. Selectivity and stability assays of SA-XV. Cytotoxicity of HCEC (a) and SNEC (b) treated with SA-XV and amphotericin B as positive control was determined by MTT assay. Hemolytic activity of SA-XV toward the human red blood cells (c). The selectivity of SA-XV towards fungus was determined by *in vitro* model of infection. HCEC were infected with *Fusarium spp.* for 6 h in presence or absence of the peptide followed by staining with propidium iodide (d) and lactate dehydrogenase assay (e). The irritation potential of the peptide was determined by hen's egg test on chorioallantoic membrane (HET-CAM) assay (f). The stability of the peptide under different conditions were determined by UPLC (g). The serum stability of the peptide was also determined (h). (* denotes $p<0.05$, ** denotes $p<0.005$, *** denotes $p<0.0005$, ns denotes not significant)

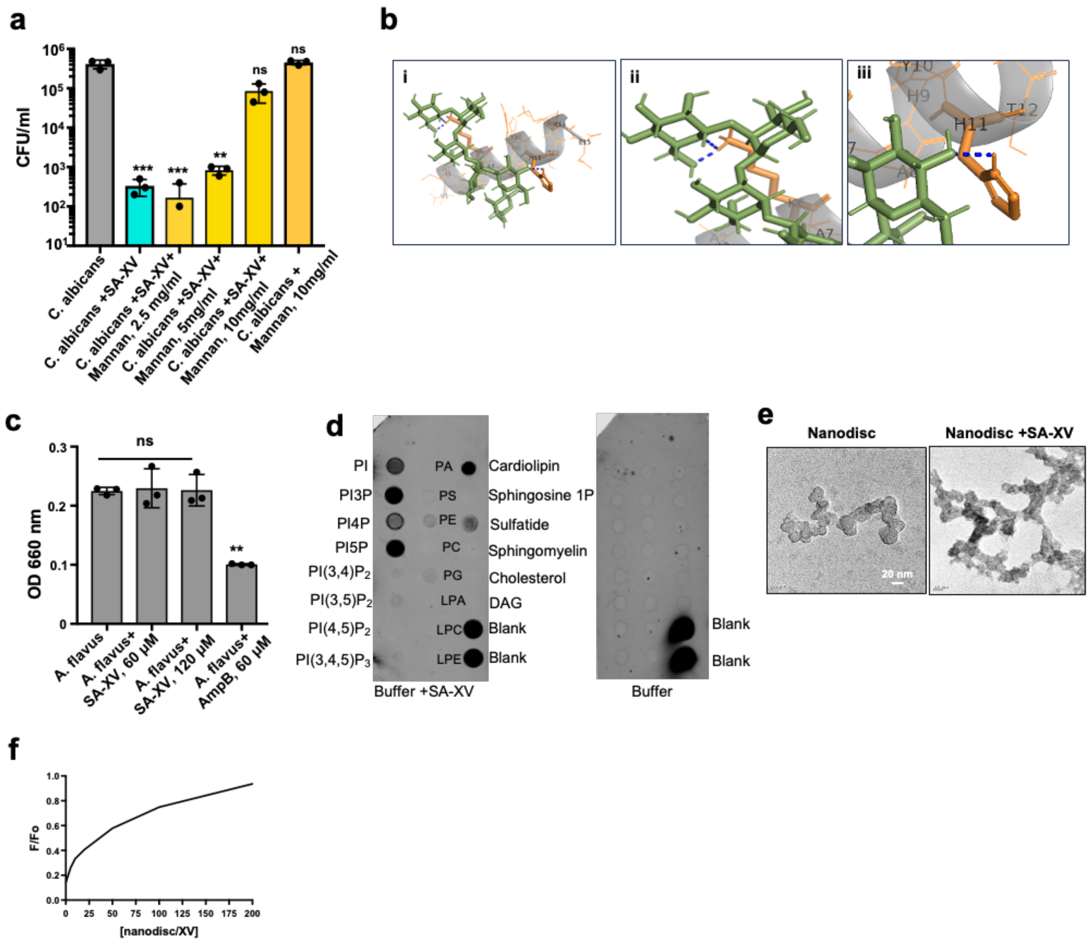

Supplementary Figure 3. Binding of SA-XV to fungal cell wall and membrane. SA-XV binds to mannan as determined by competitive assay of inhibiting fungal growth in *C. albicans* (a). Molecular docking of SA-XV to mannan done by PyMOL. Mannan is represented in green and peptide as grey cartoon form, the side chains of amino acids interacting with mannan are shown in orange. Dashed blue lines represent hydrogen bonds (b). SA-XV failed to inhibit fungal growth in *Aspergillus flavus* (c). The binding of SA-XV to several phospholipids was determined by PIP strips (d). Nanodiscs were incubated with SA-XV and imaged using TEM (e). Shift in fluorescence emission maxima of SA-XV in the presence of increasing concentrations of nanodiscs after normalization (f). (* denotes $p<0.05$, ** denotes $p<0.005$, *** denotes $p<0.0005$, ns denotes not significant)

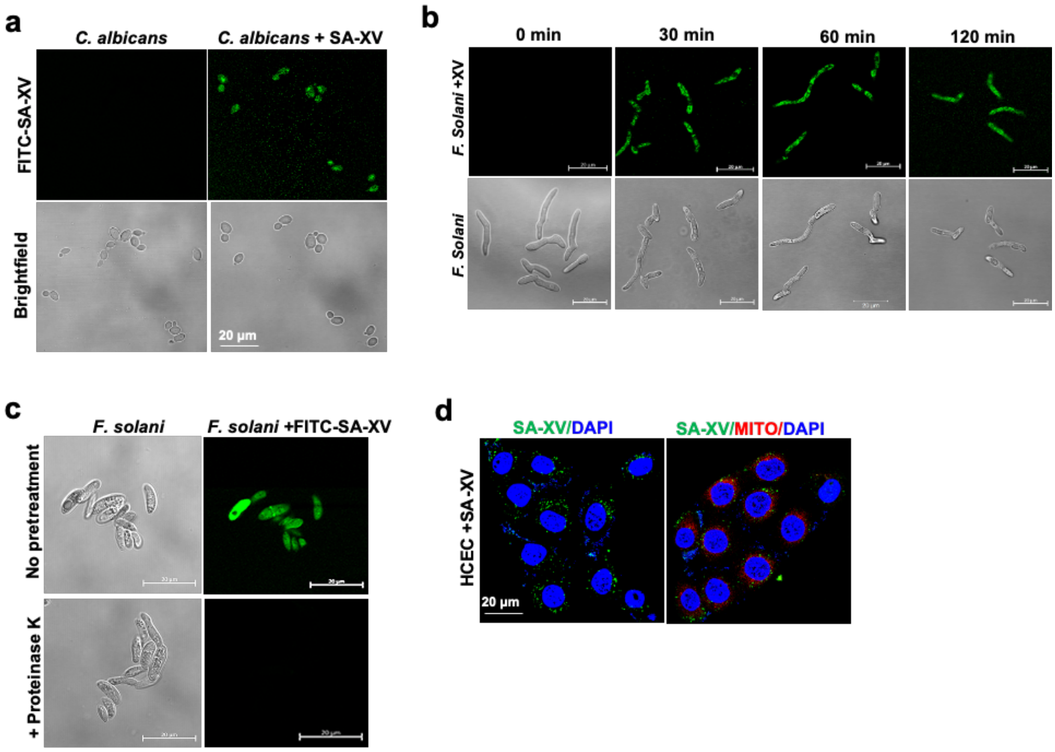

Supplementary Figure 4. Translocation of SA-XV into fungal cytoplasm. FITC-SA-XV was incubated with *C. albicans* for 2h and was found to enter into fungal cells (a). FITC-SA-XV was further incubated with F. solani for different time points and was found to enter cells by 30 min (b). Uptake of FITC-SA-XV was further monitored after removal of outer layer of glycosylated protein by proteinase K for 30 min. SA-XV failed to permeabilize when the outer layer was removed (c). The uptake of the FITC-SA-XV was also determined in HCEC by confocal microscopy, no colocalization was observed with nucleus or mitochondria (d).

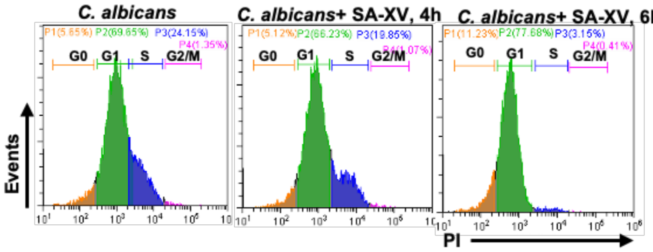

c

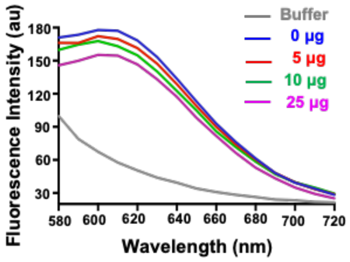

	G1 (%)	S (%)	G2/M (%)
C. albicans	69.65	24.15	1.35
C. albicans +SA-XV, 4h	66.23	19.85	1.07
C. albicans +SA-XV, 6h	77.68	3.15	0.41

b

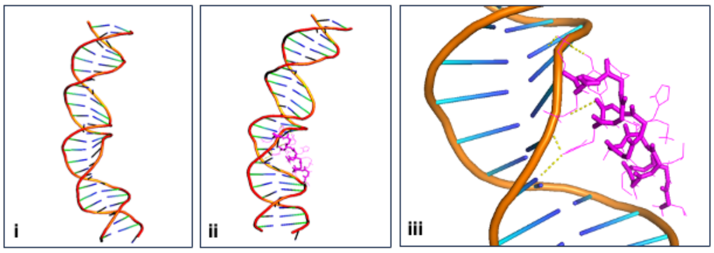

Supplementary Figure 5. Cell cycle arrest of *Candida spp.* by SA-XV was determined by flow cytometry (a). Binding of SA-XV to *Fusarium* DNA (PDB ID:4efj) was determined by molecular docking using PyMOL (b). Ethidium bromide competitive binding assay was performed by recording the fluorescence spectra from 580 – 720 nm (Ex- 535 nm) with sampling interval set to 10 nm (c).

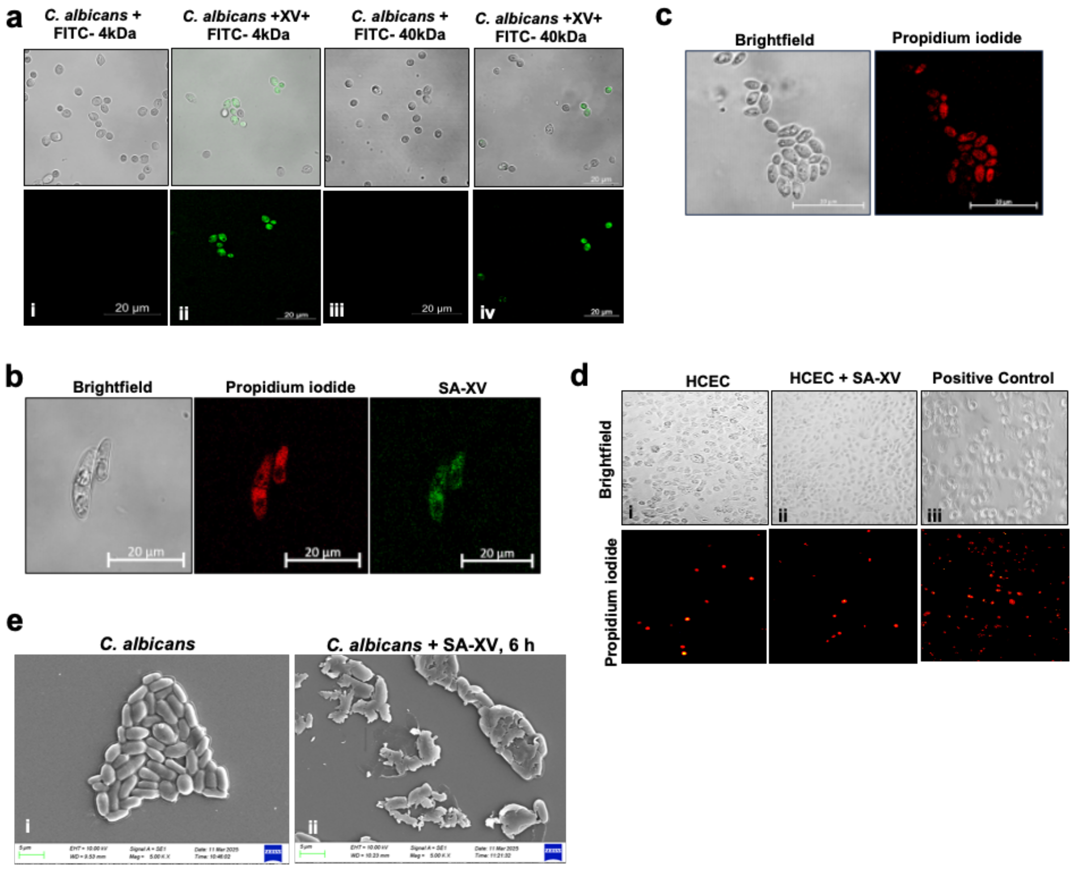

Supplementary Figure 6. SA-XV causes fungal membrane disruption. Uptake of FITC-dextran was determined in *Candida spp*. after incubation with SA-XV for 2 h (a). Uptake of propidium iodide after treatment with SA-XV for 1 h in *Fusarium spp.* (b) and *Candida spp.* (c). HCEC was treated with SA-XV for 6 h and uptake of propidium iodide was observed. Triton X-100 was used as a positive control (d). SEM images of *Candida spp.* incubated with SA-XV for 6 h (e).

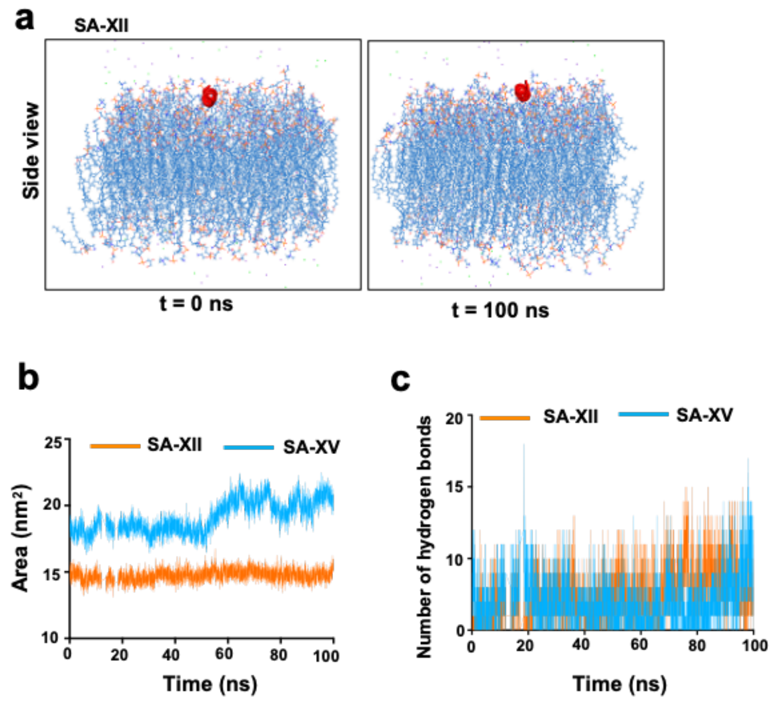

Supplementary Figure 7. Molecular dynamics simulation of SA-XII with fungal membrane. Snapshots of the top and side view of SA-XII interacting with the fungal phospholipid at 0 and 100 ns (a). The time evolution of solvent accessible surface area for SA-XV and SA-XII in fungal membrane (b). Number of hydrogen bonds formed during interaction with the fungal membrane during 100 ns simulations by SA-XV or SA-XII (c).

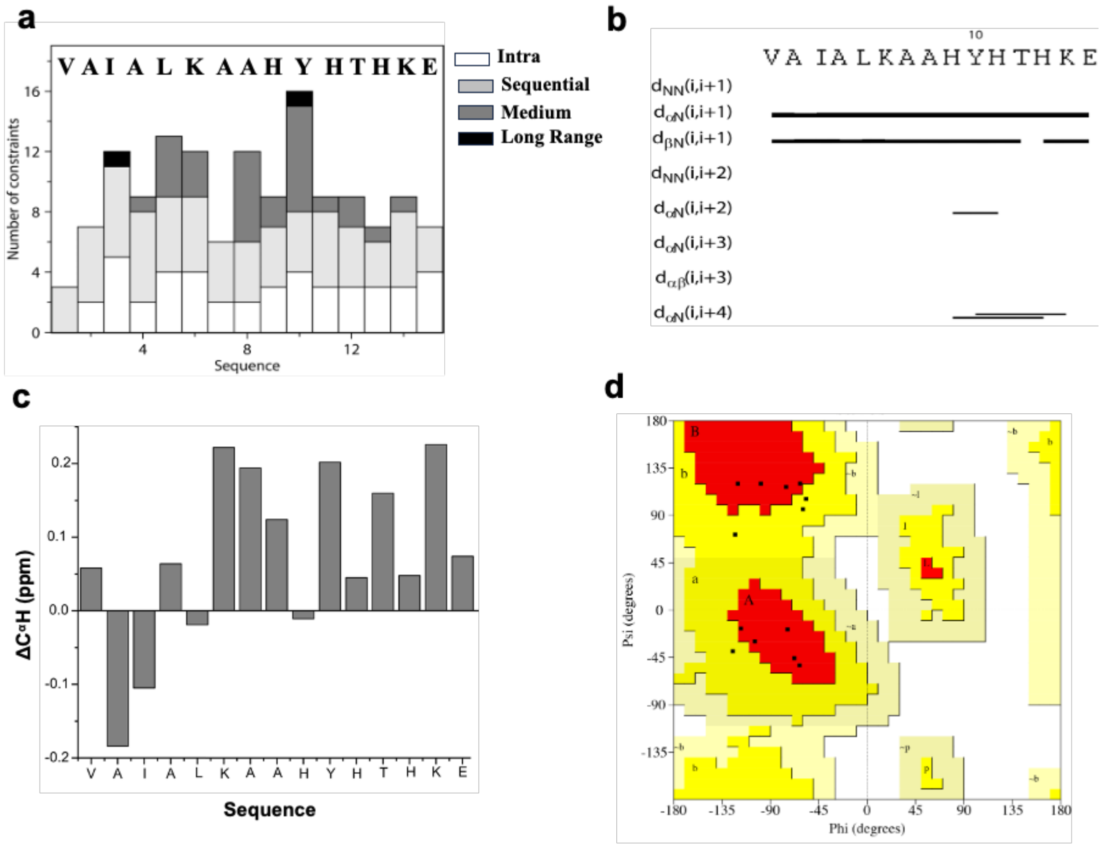

Supplementary Figure 8. Histogram depicting the number of NOEs of SA-XV in presence of 200 mM SDS micelles as a function of the residue number (a). Bar diagram showing sequential, medium range and long- range NOE connectivities of SA-XV in SDS micelles (b). The chemical shift deviation for H^{α} resonances of each residue of SA-XV from the standard random coil in SDS micelle (c). Ramachandran plot displaying the distribution of Phi (Φ) and Psi (Ψ) angles of representative NMR derived conformations of SA-XV in SDS (d).

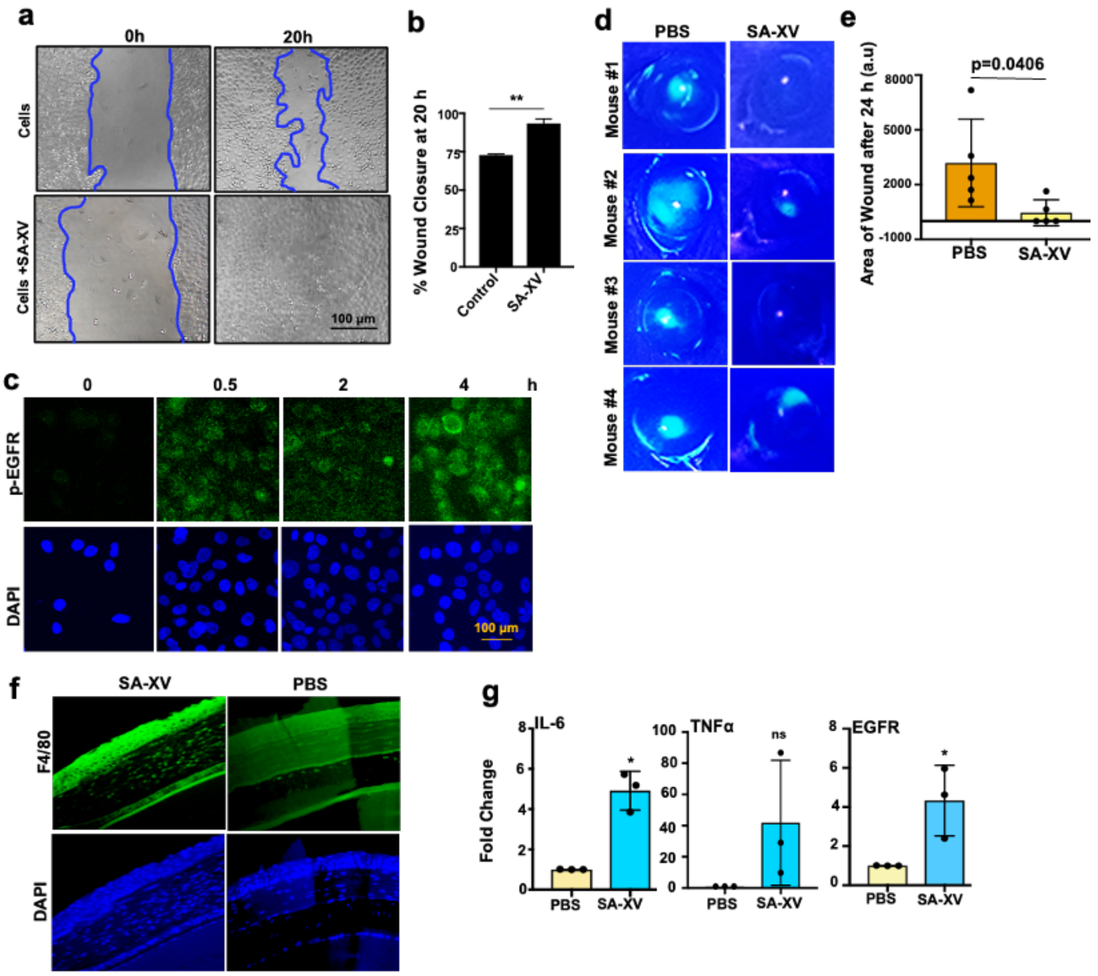

Supplementary Figure 9. SA-XV promotes cell migration and corneal wound healing. Effect of SA-XV on cell migration of human corneal epithelial cells was determined by scratch assay and imaged using microscope at 0 and 20 h post scratch. The blue line indicates the border of wounded area (a). The residual area of wound was measured by Image J and represented as percentage wound closure using bar graph (b). The cells were incubated with SA-XV for defined time points and phosphorylation of EGFR was determined by immunocytochemistry (c). The fluorescein-stained images of all mice treated with PBS or SA-XV, 24 h post-wound (d). The remaining wound area 24 h post treatment was determined by Image J and plotted graphically (e). The corneal sections of mice (n=3) treated with or without SA-XV were stained with F4/80 antibody (f). Eyes (n=3) treated with or without SA-XV after wounding were homogenized and gene expression was determined by quantitative PCR after RNA isolation and cDNA synthesis (g). (* denotes $p<0.05$, ** denotes $p<0.005$, *** denotes $p<0.0005$, ns denotes not significant)

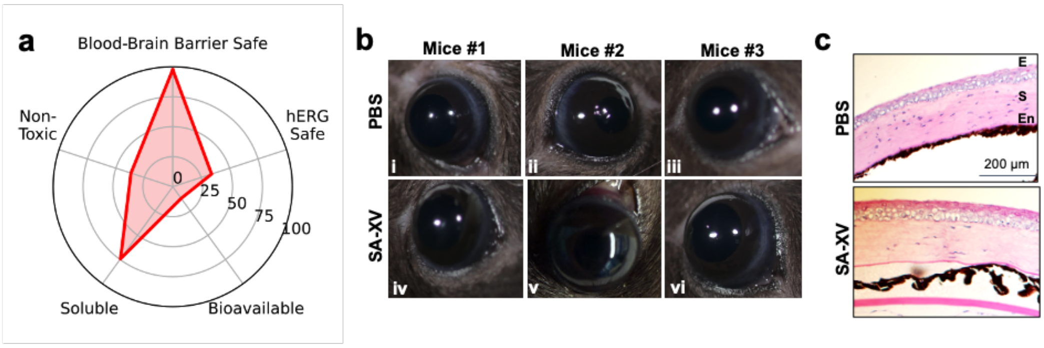

Supplementary Figure 10. Pharmacokinetic analysis of SA-XV. ADMET property prediction profiles of SA-XV suggesting the low blood brain barrier penetration and hERG toxicity indicating their lack of toxicity in human (a). *In vivo* safety assessment of the peptide visually (b) or histologically (c). No edema or hyperemia was seen in eyes treated with SA-XV. E denotes epithelium, S is stroma and En is endothelium.

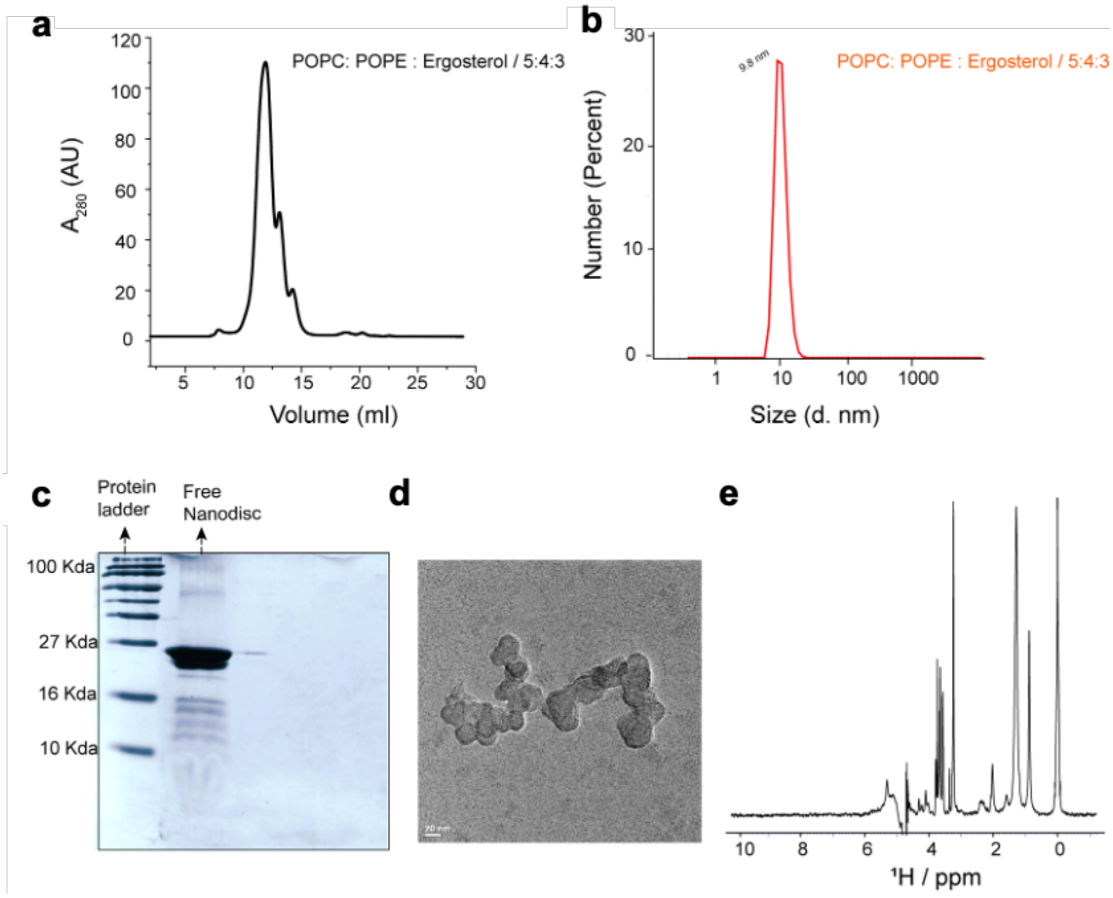

Supplementary Figure 11. Nanodisc Characterization. Characterization of free POPC/POPE/ Succinyl ergosterol 5:4:3 nanodisc through size exclusion chromatography confirms the formation of homogeneous of single sized nanodisc (a). DLS data confirms the size of the free nanodisc as 6 nm (b). SDS PAGE image of free nanodisc, single band at around 19 kDa confirms the formation of single type of MSP nanodisc (c). TEM image of free nanodisc also confirms the size of single isolated nanodisc to be around 6-10 nm. This representative image of control nanodisc is part of the experiment shown in Figure S3e, and intentionally kept same here to validate the nanodisc used in the experiment (d). 1D 1H NMR confirms the proper formation of MSP nanodisc, as no amide peaks are visible indicating MSP molecules are not in open conformation rather it perfectly wrapping around the lipid bilayers (e).

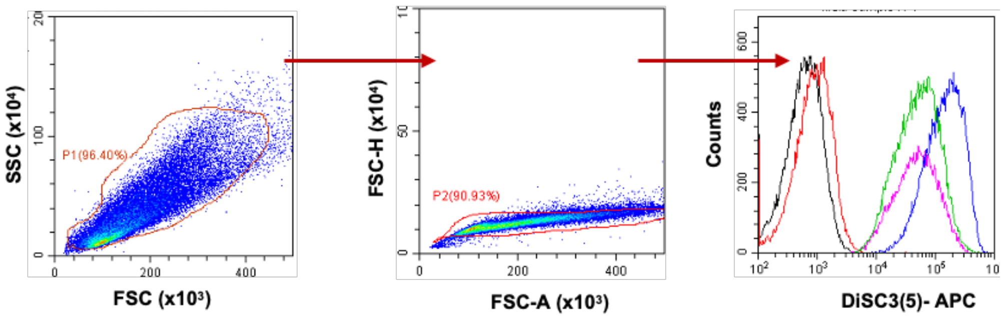

Supplementary Figure 12. Gating strategy for flow cytometry analysis for figure 3f.

Video Legend

Video S1. The peptide shown in cartoon representation and colored red is seen placed horizontally on the membrane. The lipids are shown in line or wire representation with ions shown on the extracellular side of the membrane. During the simulation, the peptide's N-terminus undergoes extension and changes the orientation from horizontal to nearly vertical. The trajectory is post-processed to remove periodic boundary conditions and centered in the simulation box.

References

1. Mishra, P., Ch, S., Ghosh, A., Kundu, S., Agarwal, R., Bhogapurapu, B., Biswas, S., and Roy, S. (2024) S100A12 inhibits Streptococcus pneumoniae and aids in wound healing of corneal epithelial cells both in vitro and in vivo. *Microbes Infect*, 105421
2. Schneider, C. A., Rasband, W. S., and Eliceiri, K. W. (2012) NIH Image to ImageJ: 25 years of image analysis. *Nat Methods* **9**, 671-675
3. Roy, S., Bonfield, T., and Tartakoff, A. M. (2013) Non-apoptotic toxicity of Pseudomonas aeruginosa toward murine cells. *PLoS One* **8**, e54245
4. Evans, B. C., Nelson, C. E., Yu, S. S., Beavers, K. R., Kim, A. J., Li, H., Nelson, H. M., Giorgio, T. D., and Duvall, C. L. (2013) Ex vivo red blood cell hemolysis assay for the evaluation of pH-responsive endosomolytic agents for cytosolic delivery of biomacromolecular drugs. *J Vis Exp*, e50166
5. Jenssen, H., and Aspmo, S. I. (2008) Serum stability of peptides. *Methods Mol Biol* **494**, 177-186
6. Padaga, S. G., Bhatt, H., Ch, S., Paul, M., Itoo, A. M., Ghosh, B., Roy, S., and Biswas, S. (2024) Glycol Chitosan-Poly(lactic acid) Conjugate Nanoparticles Encapsulating Ciprofloxacin: A Mucoadhesive, Antiquorum-Sensing, and Biofilm-Disrupting Treatment Modality for Bacterial Keratitis. *ACS Appl Mater Interfaces* **16**, 18360-18385
7. Swanson, K., Walther, P., Leitz, J., Mukherjee, S., Wu, J. C., Shivnaraine, R. V., and Zou, J. (2024) ADMET-AI: a machine learning ADMET platform for evaluation of large-scale chemical libraries. *Bioinformatics* **40**
8. Mishra, P., Ch, S., Hong, S. J., Biswas, S., and Roy, S. (2022) Antimicrobial peptide S100A12 (calgranulin C) inhibits growth, biofilm formation, pyoverdine secretion and suppresses type VI secretion system in Pseudomonas aeruginosa. *Microb Pathog* **169**, 105654
9. Hagn, F., Nasr, M. L., and Wagner, G. (2018) Assembly of phospholipid nanodiscs of controlled size for structural studies of membrane proteins by NMR. *Nat Protoc* **13**, 79-98
10. Morris, G. M., Huey, R., Lindstrom, W., Sanner, M. F., Belew, R. K., Goodsell, D. S., and Olson, A. J. (2009) AutoDock4 and AutoDockTools4: Automated docking with selective receptor flexibility. *J Comput Chem* **30**, 2785-2791
11. Laskowski, R. A., and Swindells, M. B. (2011) LigPlot+: multiple ligand-protein interaction diagrams for drug discovery. *J Chem Inf Model* **51**, 2778-2786
